# Hunting for Extremophiles: A Systematic Screening of Freshwater Microalgae for Tolerance to High-pH and High-Alkalinity Cultivation

**DOI:** 10.1021/acssuschemeng.5c09906

**Published:** 2026-02-24

**Authors:** Patrick K. Thomas, Robin Gerlach, Anita Narwani

**Affiliations:** † Department of Aquatic Ecology, 28499 Swiss Federal Institute of Aquatic Science and Technology (Eawag), 8600 Dübendorf, Switzerland; ‡ Center for Biofilm Engineering, 33052 Montana State University, Bozeman, Montana 59717, United States; § Department of Chemical and Biological Engineering, Montana State University, Bozeman, Montana 59717, United States

**Keywords:** pest management, algal bioproducts, direct air capture, bicarbonate, carbon capture, biofuels, biodiversity, circular economy

## Abstract

Microalgae hold the potential to supply sustainable food, fuel, plastics, and chemicals at commercial scales. Cultivating microalgae at extreme pH (>10) and high alkalinity provides multiple benefits, including (1) reducing the risk of contamination by undesired organisms and (2) enabling direct air capture of CO_2_, which expands the land area suitable for algae farming compared to using CO_2_ point sources alone. However, we currently have a limited understanding of which algal taxa can grow under these conditions. Therefore, we conducted a high-throughput screening of 49 freshwater microalgae strains, comprising 40 species, for their ability to grow in moderate (pH 8.5, 25 mM alkalinity), high (pH 10, 75 mM alkalinity), and extreme (pH 10, 150 mM alkalinity) cultivation environments. Our results show that moderate alkalinity tends to significantly increase algae growth (including potentially harmful strains). However, higher levels inhibited all but a small subset of green algae and cyanobacteria. Effects of salinity and alkalinity differed, indicating that they are broadly decoupled. Our results identify new industrially relevant alkaline-tolerant strains, show that algae isolated from “normal” ecosystems can be extremophilic, and suggest that future bioprospecting efforts for alkaline-tolerant algae adapted to local climatic conditions could yield additional productivity gains for the algae industry.

## Graphical abstract

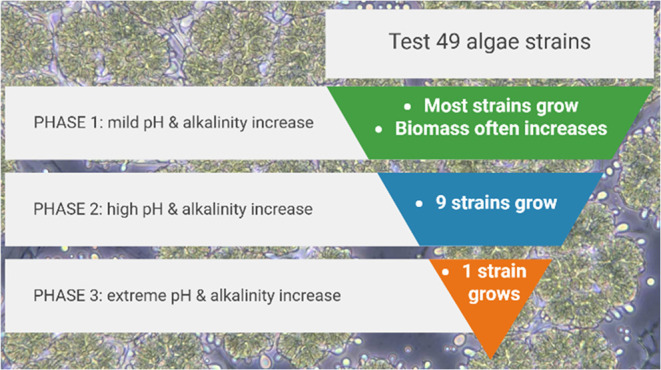

## Introduction

Microalgae farming has the potential to address several Sustainable Development Goals by provisioning much-needed protein and biobased fuels, chemicals, and polymers to society while also stimulating rural economies and more efficiently using water resources.^1^ In addition to the many foreseen benefits of developing commercial-scale algae farms for food, feed, fuel, and bioproducts, there are also several challenges that have limited the economic viability of scaling up algae cultivation.^2,3^ These challenges often involve overlapping limitations related to algal ecology and physiology, which can lead to yield instability as well as process engineering bottlenecks, which in turn can hinder the economic benefits of algae production. For example, outdoor algae cultures are subject to attack by “natural enemies” of algae, including parasites, pathogens, and grazers, as well as invasion of “weedy” algal strains.^4–6^ Engineering barriers include the need to increase harvesting and conversion efficiency,^7^ to sustainably source nutrients for algal fertilizers,^8^ and to optimize the delivery of inorganic carbon to algae in a scalable and economical manner.^9^ Proposed solutions for some of these challenges include pest management via the use of extreme pH/salinity/temperatures,^10^ the addition of biocides,^11^ manipulation of algal microbiomes,^12,13^ or the use of diverse algal polycultures that are reported to be less prone to pest outbreaks.^14,15^

Of these proposed solutions, cultivation at high pH and high alkalinity in particular holds promise, largely because it appears to have multiple cobenefits that address both biological and process engineering constraints for algae farming.^16,17^ Specifically, high-pH cultivation (i.e., pH > 10) excludes many potential biological pests from the system, as most grazers and other harmful species are not adapted to such extreme pH conditions. At the same time, using high-alkalinity ponds allows algae farming to be decoupled from point sources of CO_2_; this is because the high availability of inorganic carbon in high-carbonate-alkalinity solutions, combined with high mass transfer rates of CO_2_ from the atmosphere, facilitates direct air capture of carbon. Examples of high productivity, demonstrated in commercially relevant strains under high pH/alkalinity, include the green algae *Chlorella* sp. SLA-04 ^18,19^ and *Dunaliella salina*,^20^ the diatom *Nitzschia inconspicua* str. Hildebrandi,^21^ and several cyanobacteria ranging from well-known commercial strains like *Spirulina* ^22^ to model strains like *Synechocystis* PCC 6803 ^20^ and a newly isolated strain of *Cyanobacterium* from an alkaline lake.^23^

Despite the potential of high-pH and high-alkalinity cultivation to enhance the reliability and productivity of sustainable algae farming, only a relatively limited number of algae strains have been tested for their ability to grow under alkaline conditions, leaving us with a minimal understanding of the algal taxa that may tolerate such cultivation environments. Moreover, while researchers have identified several economically beneficial alkaliphilic strains, we currently lack an understanding of whether potentially harmful algae strains could also grow in high-pH and high-alkalinity ponds. In this context, “harmful” refers to any microalgae species that would cause economic harm to algae farms (e.g., those noted in Table 1). For example, toxin-producing cyanobacteria such as *Microcystis* and *Planktothrix*, which form harmful blooms in natural aquatic systems,^24^ could threaten the safety of algae-based food crops, while harmful mixotrophic algae such as *Poterioochromonas* can directly consume smaller algal taxa, thus decimating crops.^25^

**Table 1:** Algae Strains Used in This Study

broad group	species	culture collection	strain ID
chrysophyte	^a^ *Poterioochromonas malhamensis*	CCAP	933/1C
cryptophyte	*Cryptomonas* sp.	NIVA	3/09
cyanobacteria	^a^ *Anabaena planctonica*	NIVA-CYA	651
cyanobacteria	^a^ *Aphanizomenon flos-aquae*	NIVA-CYA	681
cyanobacteria	^a^ *Microcystis aeruginosa*	Eawag isolate	Greifensee
cyanobacteria	^a^ *M. aeruginosa*	PCC	7806
cyanobacteria	^a^ *Planktothrix rubescens*	NIVA-CYA	619
cyanobacteria	*Synechocystis* sp.	PCC	6803
cyanobacteria	*Synechococcus elongatus*	Eawag isolate	GM48
diatom	*Cyclotella meneghiniana*	SAG	1020-1a
diatom	*Fistulifera saprophila*	Eawag isolate	GM16
diatom	*Fragilaria capucina*	CCAC	2678B
diatom	*Fragilaria crotonensis*	Eawag isolate	GM2
diatom	*Fragilaria mesolepta*	Eawag isolate	GM15
dinoflagellate	*Cystodinium* sp.	SAG	59.87
eustigmatophyte	*Nannochloropsis limnetica*	SAG	18.99
eustigmatophyte	*Nannochloropsis oculata*	SAG	38.85
green algae	*Ankistrodesmus falcatus*	SAG	202-2
green algae	*Botryococcus braunii*	SAG	30.81
green algae	*B. braunii*	CCAP	807/1
green algae	*B. braunii*	CCAP	807/2
green algae	*B. braunii*	NIES	2199
green algae	*B. braunii*	Eawag isolate	PT1
green algae	*B. braunii*	N/A	Showa
green algae	*Chlamydomonas reinhardtii*	CC	1690
green algae	*C. reinhardtii*	CC	3060
green algae	*Chlorella vulgaris*	SAG	211-11b
green algae	*Cosmarium botrytis*	SAG	136.8
green algae	*Desmodesmus abundans*	Eawag isolate	GM26
green algae	*Desmodesmus armatus*	Eawag isolate	L t0
green algae	*Kirchneriella subcapitata*	SAG	12.81
green algae	*Lagerheimia hindakii*	SAG	11.92
green algae	*Lagerheimia subsalsa*	Eawag isolate	GM37
green algae	*Medakamo hakoo*	NIES	4000
green algae	*Messastrum gracile*	Eawag isolate	GM45
green algae	*Micractinium pusillum*	CCAP	231/1
green algae	*Oocystella heteromucosa*	Eawag isolate	GM35
green algae	*Oocystis solitaria*	SAG	83.8
green algae	*Oocystis* sp.	Eawag isolate	L t0
green algae	*Pandorina unicocca*	Eawag isolate	GM17
green algae	*Pediastrum boryanum*	SAG	87.81
green algae	*P. boryanum*	Eawag isolate	GM20
green algae	*Pediastrum duplex*	SAG	261-2
green algae	*P. duplex*	Eawag isolate	GM22
green algae	*Scenedesmus acuminatus*	SAG	38.81
green algae	*Scenedesmus armatus*	Eawag isolate	GM28
green algae	*Scenedesmus ellipticus*	Eawag isolate	GM25
green algae	*Staurastrum punctulatum*	SAG	679-1
green algae	*Tetraedron minimum*	SAG	44.81

a Denotes strains designated as potentially harmful for commercial algae farming.

To help overcome these knowledge gaps, we conducted a systematic screening of 49 algae strains, comprising 40 species, from taxonomically diverse groups of phytoplankton in order to broaden our understanding of high-pH and alkalinity tolerance in algae. This bioprospecting effort focuses specifically on freshwater microalgae because their tolerance to high pH and alkalinity is largely unknown at present. The main goals of this effort are (1) to identify novel algae strains capable of growing in high-pH and high-alkalinity conditions, which could be used in commercial farming, (2) to test whether a set of reportedly harmful algae species can also grow in high-pH and high-alkalinity conditions, and (3) to disentangle effects of salinity versus pH and alkalinity on algal growth. We expected only a fraction of the strains tested to be productive in these extreme conditions, but we also expected to discover additional alkaline-tolerant strains that could be of value for industrial algae cultivation for direct air capture of carbon dioxide into value-added bioproducts.

## Materials and Methods

### Algae Cultures

The Aquatic Ecology Department of Eawag maintains a large collection of microalgal strains representing a broad array of taxa from unique environmental origins. This collection presented a unique opportunity to bioprospect for high-pH and high-alkalinity tolerance across much of the freshwater phytoplankton tree of life. The cultures selected for this experiment range from recent isolates from Swiss lakes to many standard “type strains” obtained from culture collections. This allows us to test not only laboratory-adapted model strains but also those more recently isolated from natural settings. With the exception of a few brackish strains (e.g., *Nannochloropsis oculata*), the microalgae used are assumed to be adapted primarily to freshwater (i.e., this study does not include strictly marine strains). The strains used in the experiment are listed in Table 1. Before the experiment, cultures were maintained at 20 °C in COMBO medium with double the concentration of all nutrients described in the original medium recipe;^26^ this medium is hereafter referred to as 2X COMBO. This common medium was used for all strains, as it is designed to allow growth of many distinct algal taxa (e.g., diatoms, green algae, cyanobacteria); in terms of major nutrients, 2X COMBO contains 2000 μM N, 100 μM P, and 200 μM Si (see Kilham et al.^26^ for full recipe). While higher concentrations of Si (>1 mM) can inhibit nondiatom algae, the concentration used here is not expected to cause inhibition, as COMBO is shown to accommodate growth of numerous nondiatom strains,^26^ and 200 μM is within the range of background Si concentrations observed in freshwater systems.^27^

### Experimental Design

The primary factor that was experimentally manipulated in this experiment (apart from the algal strain identity) was the medium type. The study is organized into three phases, with each phase exposing algae to a growth medium with a higher level of pH/alkalinity (as well as salinity) than the previous one. The rationale for this phased approach was that stepwise acclimation may reveal tolerances that cannot be observed by directly subjecting algae to more extreme conditions. In each phase, the three different media types tested were (1) 2X COMBO, which served as a positive control; (2) 2X COMBO with added NaCl at increasing levels from phase to phase, which served to distinguish salinity tolerance from pH/alkalinity tolerance; and (3) 2X COMBO with added NaHCO_3_ and/or Na_2_CO_3_ to identify high pH/alkalinity tolerance. The salinity treatments match the mass concentration of salinity contributed by the added carbonate salts; this results in a 44% higher molar concentration of NaCl versus carbonate salts in each phase (e.g., 2.1 g/L NaCl equals 35.9 mM, while 2.1 g/L NaHCO_3_ equals 25 mM). Table 2 describes the media treatment levels used for each of the three phases. pH was measured with Mettler Toledo SevenDirect SD20. In Phase 1, only NaHCO_3_ was used to add alkalinity (and moderately increase the initial pH to near 8.5). In Phases 2 and 3, an equimolar mixture of NaHCO_3_ and Na_2_CO_3_ was used as this increases the initial pH to the desired level (near 10) without requiring addition of NaOH or other basic solutions (i.e., the buffering capacity remains the same as if only NaHCO_3_ were added, but this equimolar mixture eliminates the need to add liquid base solution which would slightly dilute the medium). The total alkalinity increase due to these sources is described as added carbonate alkalinity = [HCO_3_^–^] + 2 × [CO_3_^2–^].

**Table 2:** Experimental Treatments Used in Each Phase

phase	control treatment	NaCl treatment	high-pH and high-alkalinity treatment
Phase 1: acclimation	2X COMBO (pH 7.5)	2.1 g/L NaCl (pH 7.5)	2.1 g/L NaHCO_3_ (pH 8.49; 25 mM added carbonate alkalinity)
Phase 2: high-alkalinity screening	2X COMBO (pH 7.5)	4.75 g/L NaCl (pH 7.5)	2.1 g/L NaHCO_3_ and 2.65 g/L Na_2_CO_3_ (pH 10.09; 75 mM added carbonate alkalinity)
Phase 3: extreme alkalinity screening	2X COMBO (pH 7.5)	9.5 g/L NaCl (pH 7.5)	4.20 g/L NaHCO_3_ and 5.30 g/L Na_2_CO_3_ (pH 10.02; 150 mM added carbonate alkalinity)

With the exception of the above media manipulations, each phase used identical methods for the cultivation of microalgae. Specifically, 900 μL of medium per well was dispensed into sterile 48-well plates, and 100 μL of algae culture was added to yield a total volume of 1 mL per well, with 4 replicates per treatment. The phased acclimation approach was carried out as follows: for Phase 1, the 100 μL algal inoculum came from 50 mL flask precultures; for Phase 2, the 100 μL algae came directly from the corresponding media treatment wells at the end of Phase 1; similarly, the 100 μL algae to start Phase 3 came directly from the corresponding Phase 2 wells. This was done to ensure that the algae populations acclimated to a given phase would be directly tested for their tolerance to the subsequent and progressively harsher environment. It also mimics, in a microcosm environment, the act of transferring algae cultures via a 10% v/v dilution, as might be done when commercial cultivation is scaled up from smaller to larger raceway volumes, and it maintains a constant volume proportion of new medium across treatments. A potential limitation of this sequential transfer approach is that while the wells were always inoculated with the same volume of algae culture (100 μL; 10% by volume), they were not equalized in terms of initial algal biomass concentration. Therefore, the reader should be aware that differences in initial densities across both strains and media treatments could influence the resulting growth parameters. In rare cases, a strain grew and then declined before transfer to the next phase, potentially affecting subsequent growth. However, these strains in decline also often grew well in the following phase despite their decline (e.g., see *Synechocystis* PCC 6803 in Figures S1 and S2). Also note that due to within-population variability, a declining culture may still contain a minority of alkaline-tolerant cells; this is why even poor-performing strains were transferred from Phase 1 to Phase 2. The durations of experimental phases were 7 days (Phase 1); 15 days (Phase 2); and 10 days (Phase 3). The duration of Phase 1 was chosen to provide a week-long physiological acclimation period to mildly increased pH and alkalinity, while the durations of Phase 2 and 3 were chosen to allow all cultures to reach carrying capacity in a given treatment (i.e., ensuring maximum fluorescence values reflected the biomass concentration upon stabilizing at the stationary phase). Only the 10 best-performing strains from Phase 2 were used in Phase 3; these were all relatively fast-growing and thus reached a stationary phase earlier, resulting in a shorter duration than in Phase 2.

In all phases, algae were grown in a Memmert ICP 700 incubator set to 24 °C with cool white fluorescent lights set to 14 h:10 h light:dark cycle and 98.2 ± 14.3 SD μmol photons m^–2^ s^–1^. All plates were covered with Breathe Easy membranes (Diversified Biotech) to allow for gas exchange and minimize evaporation. Growth was tracked using *in vivo* fluorescence (recorded in terms of relative fluorescence units, RFUs) as a proxy for the total algal biomass concentration; measurements were taken every 24–48 h. Chlorophyll a fluorescence was measured directly in the wells of the microplates for all experimental units with excitation/emission at 445/685 nm, using a Biotek Cytation 5 plate reader. This general method is described by Van Wagenen et al.^28^ and has been used in numerous studies to track algal growth, although with minor differences in excitation/emission wavelengths used.^29–34^ Similarly, changes in phycocyanin concentrations in the wells were estimated using excitation/emission wavelengths of 586/647 nm (i.e., wavelengths yielding the greatest specificity to phycocyanin^35,36^ ); this was used as a proxy to track the growth of cyanobacteria. Fluorescence was chosen over optical density as a proxy for algal biomass concentration because it is more specific to photosynthetically active algal biomass (i.e., it reduces the effect of changes in nonphotosynthetic bacterial abundance, cellular debris, or mineral precipitates) and is significantly more sensitive than optical density when growing algae from low densities.^28^ However, the reader should note that, as with any biomass proxy, fluorescence should not be interpreted as a perfect substitute for direct biomass concentration measurements (e.g., ash-free dry weight). Measuring the ash-free dry weight becomes logistically prohibitive for high-throughput screening studies such as this. Additional caveats of using well plates should be noted; first, carbon limitation is possible in treatments without added alkalinity. This could influence growth rates, and therefore, these treatments are not meant to be representative of algal cultivation with CO_2_ sparging. Similarly, the small volumes in the microplates prevent continuous measurement or control of pH; therefore, we can only report initial pH values. We expect treatments without added alkalinity to experience an overall increase in pH and greater daily pH swings following the light/dark cycle than higher-alkalinity treatments due to differences in buffering capacity, as observed in previous work.^18,23,37–39^

### Statistical Analysis

The two main metrics used to assess treatment effects in this study are (1) the maximum fluorescence attained over the course of an experimental phase, which serves as a proxy for the maximum algal biomass concentration, and (2) the growth rate over time. Each of these values was calculated for each experimental unit (*n* = 4 replicates for each combination of strain/treatment/phase). It should again be noted that max. fluorescence can be affected by both algal biomass concentration and per-cell chlorophyll, thus representing a relative (and not absolute) proxy of peak photosynthetically active biomass, and the initial density in each phase may affect both the growth rate and maximum biomass concentration. Effects of treatments on max. fluorescence are shown in figures in terms of the mean % change effect caused by a treatment relative to the average max. fluorescence of the control as well as the 95% confidence intervals of the effect (i.e., the confidence intervals indicate variability in the treatments but not the control). All analyses were performed in R version 4.4.3.^40^ All growth curves used to calculate summary growth data are provided in the Supporting Information (Figures S1–S3).

We assessed several complementary methods for estimating growth rates to ensure that the maximum specific growth rates obtained were realistic and not biased by a particular model fit. Specifically, we tested exponential, logistic, and Gompertz model fits using v0.8.4 of the R package “growthrates”,^41^ a suite of models in v1.2 of the R package “growthTools”^42^ in which the best of five models applied is selected by AICc scores, as well as the “manual approach” of calculating growth using the formula μ_max_ = [ln­(fluorescence day 4/fluorescence day 0)]/(4 days); this period was chosen as the first 4 days best captured exponential growth when considering all strains and environments. The different methods yielded similar median growth rate estimates (Figure S4), and growth rates tended to be highly correlated (Pearson’s *r* up to 0.96), with the exception of the logistic and Gompertz models, which often overestimated growth, resulting in many outliers with unrealistic growth rate estimates (Figures S4 and S5), making these models inappropriate overall. In sum, we find that the choice of the growth rate estimation method among the three remaining suitable methods does not substantially affect inferences drawn from our data. In the main text, we show the growth rate estimates obtained using the “growthTools” package, as this method allows for the incorporation of lag phases (observed in ca. 8% of cases), which yields a slightly more accurate estimate of exponential growth after a lag compared to the other options, and has the highest mean model *R* ^2^ value (Figure S6).

As initial densities varied substantially in the acclimation period (Phase 1), we do not assess growth rates during this time, as these density-dependent effects likely influenced growth rates. However, initial densities for Phases 2 and 3 are meant to represent approximately 10% of the carrying capacity for each strain × environment combination (due to 10% v/v transfer of stationary phase cultures), which makes growth rate comparisons across strains and treatments more appropriate. Effects of initial densities are explored further in Figure S7; in summary, we observe no overall significant effect of variation in initial density on growth rates in Phase 2, although the effects of initial densities differ among the media types (i.e., increasing initial density had no effect on the pH and alkalinity treatments but a slightly negative effect on controls).

Welch’s ANOVAs were used to test for significant differences among the three treatments for each strain in each phase, as these allow for heterogeneity of variances, which was observed throughout the data. Games–Howell post-hoc tests (analogous to Tukey tests) were performed to test for differences between means, as they also allow for heterogeneous variances and control for type 1 error. We tested the ability of salinity responses to predict alkalinity responses using a linear model with the experimental phase as a covarying fixed effect; the 95% confidence intervals of the slope estimate were then assessed to determine whether the slope differed from 1. Here, a slope of 1 would indicate a 1:1 relationship and that salinity tolerance drives alkalinity tolerance, whereas a slope differing from 1 would indicate decoupling of the two responses. In the Results section, we show the data for each strain in order of increasing performance to highlight strains with the clearest potential for high-pH and high-alkalinity growth in each phase; see Figures S8–S10 for data arranged alphabetically by strain. All data and code used are publicly available on Zenodo at https://doi.org/10.5281/zenodo.18625807.

## Results

### Phase 1: Acclimation to Moderate Alkalinity Conditions

In the first experimental phase, the addition of alkalinity as NaHCO_3_ had a generally positive effect on the algal biomass concentration (Figure 1). Twenty-one of the 49 strains (43%) reached a significantly higher maximum fluorescence value than their respective controls (see Table S1 for the Games–Howell test statistics); moreover, 19 of these strains had a 25% or greater increase in biomass relative to controls, and 4 strains had a biomass increase of over 100% with NaHCO_3_ addition. Of the remaining strains, 15/49 (30.6%) showed no significant effects of NaHCO_3_ addition, while 13/49 (26.5%) had significantly reduced biomass in terms of *in vivo* fluorescence under the moderate increase in pH/alkalinity (25 mM carbonate alkalinity).

**Figure 1:**
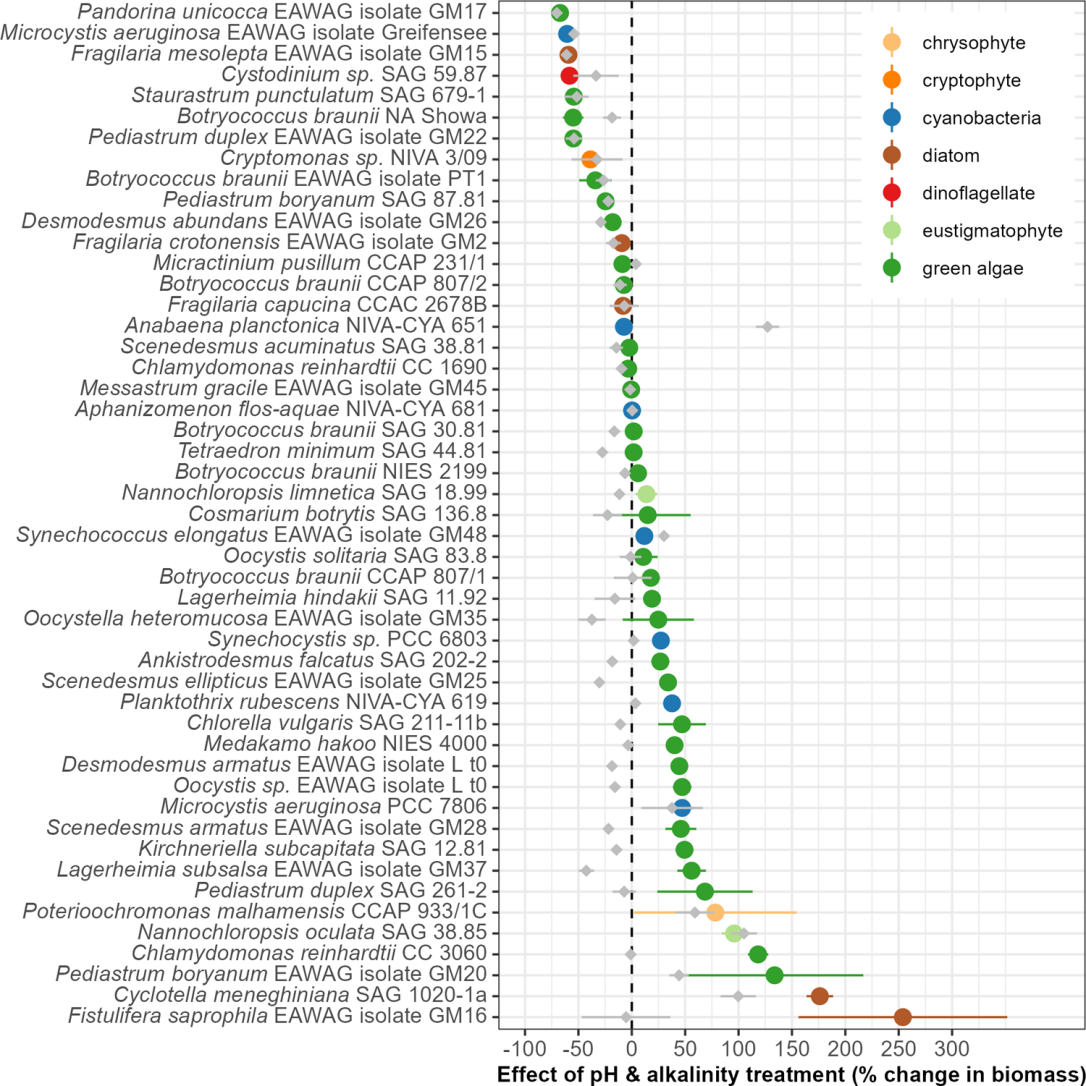
Effects of pH and alkalinity treatments on algal biomass concentration during Phase 1 (i.e., during the 7-day acclimation phase to a mild increase in initial pH and alkalinity: pH 8.5 and 25 mM alkalinity added as carbonates). Colored circles and the corresponding lines represent the mean and 95% confidence interval of the pH/alkalinity treatment effect in the form of the maximum biomass production estimated as effect size = (treatment max. fluorescence – mean control max. fluorescence)/(mean control max. fluorescence). Gray diamonds and lines show the mean and 95% CI effect of salinity (2.1 g/L NaCl during Phase 1) for each strain. Values above zero indicate a positive effect of the treatment relative to the control; zero indicates no effect; and negative values indicate a detrimental effect of the pH/alkalinity (or salinity) treatment.

Compared to the effects of pH/alkalinity, the magnitude of salinity treatment (2.1 g/L NaCl) effects on algal growth was relatively low (i.e., gray diamonds in Figure 1), especially when compared to algae strains with highly positive responses to pH/alkalinity. Although the effects of NaCl addition at 2.1 g/L were weaker overall than the effects of 2.1 g/L NaHCO_3_ addition, there was a tendency toward negative effects, with 23/49 strains significantly inhibited in growth and only 6/49 showing higher growth with NaCl versus the control (Table S1). While several strains had positive responses to both NaHCO_3_ and NaCl of roughly equal magnitude (e.g., *N. oculata* and *C. meneghiniana*), many others exhibited drastically different responses to the two treatments, and in the case of *A. planctonica*, a strong positive response to NaCl but no effect of NaHCO_3_. In total, 25/49 strains had significantly higher max. fluorescence with alkalinity versus salinity, 19/49 had equivalent effects of each, and only 5 had higher max. fluorescence with added salinity compared to added alkalinity. A linear regression indeed shows that while NaCl responses are significantly related to NaHCO_3_ responses in Phase 1, they only explain 24% of the variance in NaHCO_3_ response effects (*F*_1,47_ = 16.4, *p* = 0.0002); see Figure 2 for the salinity–alkalinty response relationships across all three phases. In other words, data from this portion of the experiment show that algal responses to moderately alkaline conditions tend to be decoupled in magnitude from their responses to moderately saline conditions. These results also show that moderate bicarbonate addition often (but not always) has a significant growth-stimulating effect that can be observed across a suite of taxonomically distinct algae such as chrysophytes, cyanobacteria, eustigmatophytes, green algae, and diatoms.

**Figure 2:**
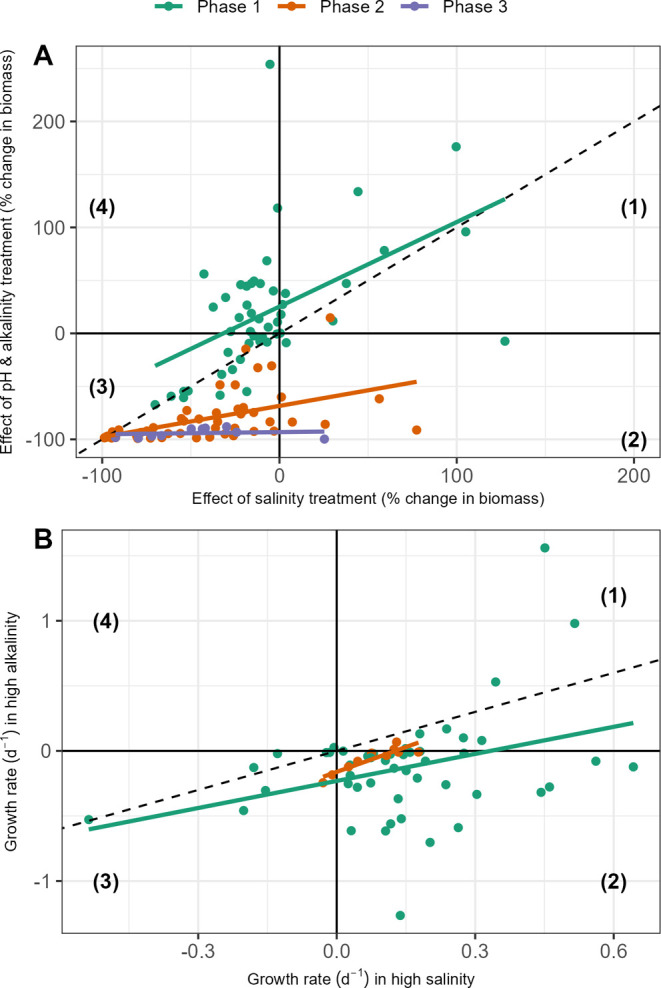
Relationship between salinity effect size and pH/alkalinity effect size and on max. fluorescence (A), and the relationship between growth rate in salinity versus pH/alkalinity treatments (B), grouped by each experimental phase. As in previous figures, effects on biomass concentration are calculated as effect size = (treatment max. fluorescence – mean control max. fluorescence)/(mean control max. fluorescence). Effect sizes (A) and growth rates (B) can each be split into four quadrants: (1) positive for both treatments; (2) positive for salinity but negative for alkalinity; (3) negative for both; and (4) negative for salinity but positive for alkalinity. In Phase 1, the decoupling of alkalinity and salinity results in more positive effects of alkalinity, while the inverse is true for the more extreme conditions in Phases 2 and 3. Points show means for each algal strain and phase; solid lines show linear model fits by phase; and the dashed lines show a 1-to-1 relationship, which would indicate equal effects of salinity and alkalinity. Slope estimates from multiple regression, including phase as a covariate, are 0.50 (95% CI: 0.30–0.69) in (A) and 0.70 (95% CI: 0.21–1.2) in (B).

While there is a clear growth enhancement of several industrially relevant production strains (e.g., *N. oculata*, *C. vulgaris*, and *C. reinhardtii*) in Phase 1, there is also a growth enhancement of certain potentially toxic cyanobacteria (e.g., *P. rubescens* and *M. aeruginosa*) as well as the mixotrophic grazer *P. malhamensis*, which is a widespread and destructive pest in algae farms.^25^ There is also evidence for within-species variation in tolerance to high pH/alkalinity; the 6 unique strains of the hydrocarbon-rich alga *B. braunii* which we tested in this study have distinct responses to increased pH/alkalinity, ranging from minor increases (e.g., strain CCAP 807/1) to drastic decreases (e.g., strain Showa) in growth (Figures 1 and S1; see also Figure S8 for side by side comparisons within genus/species). Additional evidence exists for within-species differences in responses; e.g., the two unique strains of *P. duplex* have diverging responses to the alkalinity treatment in Phase 1, as do the two strains of *P. boryanum*. Within-genus differences are also noteworthy in *Desmodesmus*, *Scenedesmus*, and *Nannochloropsis*.

### Phase 2: Screening for Tolerance to High pH and Alkalinity Levels

In contrast to the observed results for Phase 1, nearly all strains (47/49) had reduced maximum biomass concentration compared to the controls when subjected to high pH and alkalinity in Phase 2 (15 days of growth at pH 10 and 75 mM carbonate alkalinity; inoculated with a 10% v/v transfer from Phase 1 cultures; Figure 3). Only *Synechocystis* PCC 6803 had greater biomass in the high-pH/alkalinity treatment than in the control, while all but one strain (with no significant effect) had significant decreases (Table S2). Analysis of growth rates shows that approximately 18% of the strains (9/49) had positive average growth rates under these conditions (Figure 4). Within-genus and within-species differences in growth rates were noteable for some taxa (e.g., *Desmodesmus* and *P. duplex*, respectively; Figure S10). The consistent differences between the salinity and the pH/alkalinity treatments on both biomass concentration and growth rates again indicate that, in most cases, the impact on growth is likely due to pH/alkalinity *per se* rather than due to the increase in salinity, which is concomitant with increased alkalinity. For a relatively small subset of strains, however, we do observe very similar responses to salinity and pH/alkalinity (see, e.g., *P. unicocca*, *F. capucina*, and *S. punctulatum*). In contrast to Phase 1, all potentially toxic or harmful “pest” species were severely inhibited and incapable of growth under the high-pH/alkalinity conditions of Phase 2. Instead, species with positive growth rates included industrially important strains of green algae from the family Scenedesmaceae (*Desmodesmus* and *Scenedesmus*) and the cyanobacteria (*Synechocystis* and *Synechococcus*).

**Figure 3:**
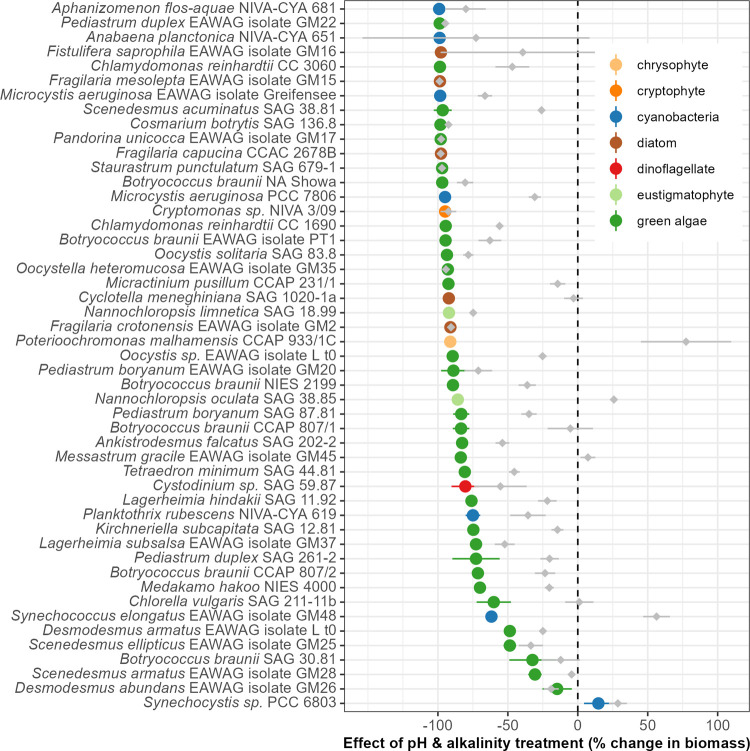
Effects of the pH and alkalinity treatment on algal biomass concentration during Phase 2 (i.e., the 15-day screening in high pH and alkalinity, pH 10 and 75 mM of alkalinity added as carbonates). Colored circles and the corresponding lines represent the mean and 95% confidence interval of the pH/alkalinity treatment effect in the form of the maximum biomass production estimated as effect size = (treatment max. fluorescence – mean control max. fluorescence)/(mean control max. fluorescence). Gray diamonds and lines show the mean and 95% CI effects of salinity (4.75 g/L NaCl during Phase 2) for each strain. Values above zero indicate a positive effect of the treatment relative to control, zero indicates no effect, and negative values indicate a detrimental effect of the pH/alkalinity (or salinity) treatment.

**Figure 4:**
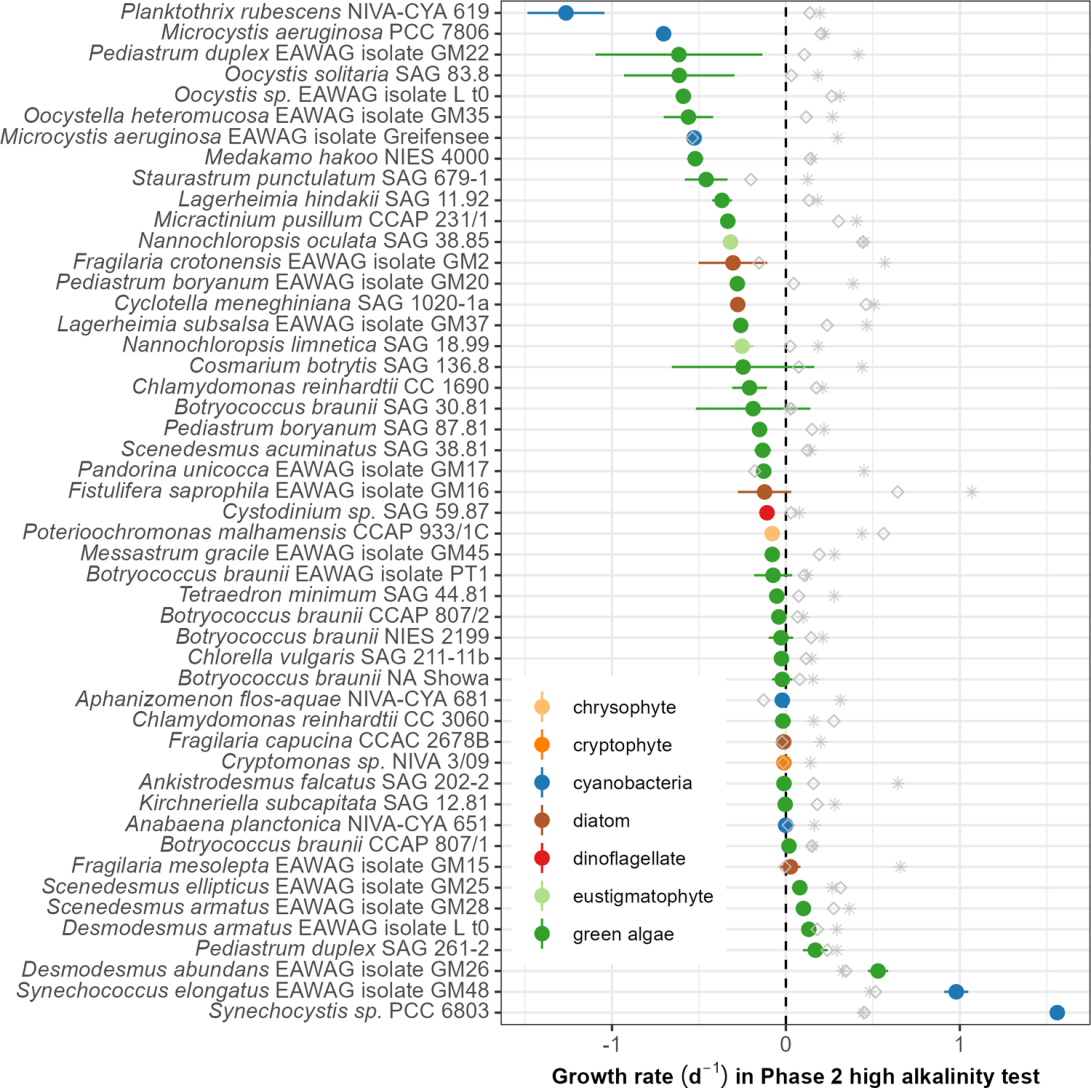
Growth rates of each algal strain in the high-pH and high-alkalinity screening (Phase 2, 15-day screening at pH 10 and 75 mM of alkalinity added as carbonates), estimated using the R package “growthTools”. Colored circles and the corresponding lines represent the mean and 95% confidence interval of the growth rate in high pH and alkalinity; gray diamonds show the average growth rates of the salinity treatments (4.75 g/L NaCl), and asterisks show the average growth rates in the control treatment.

### Phase 3: Screening for Tolerance to Extreme pH and Alkalinity Levels

Only the 10 best-performing strains in Phase 2 were transferred to the extreme conditions of Phase 3. The extreme pH and alkalinity levels in Phase 3 reduced biomass by over 80% for all strains (Figure 5), and only one strain (*D. abundans*) was able to maintain a positive growth rate (Figure 6). Again, the effects of pH/alkalinity and salinity were decoupled; although salinity generally had a negative effect compared to the control, it was much weaker than the pH/alkalinity effect. In the case of *Synechocystis* PCC 6803, the salinity appeared to induce a mild increase in maximum biomass.

**Figure 5:**
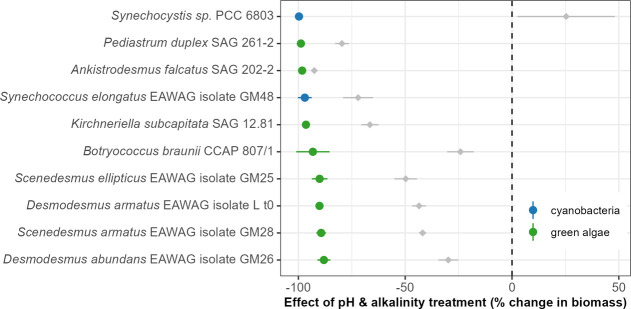
Effects of the pH and alkalinity treatment on algal biomass concentration during Phase 3 (i.e., the 10-day screening in extreme pH and alkalinity, pH 10 and 150 mM of alkalinity added as carbonates). Colored circles and corresponding lines represent the mean and 95% confidence interval of the pH/alkalinity treatment effect in the form of maximum biomass production estimated as effect size = (treatment max. fluorescence – mean control max. fluorescence)/(mean control max. fluorescence). Gray diamonds and lines show the mean and 95% CI effect of salinity (9.5 g/L NaCl during Phase 3) for each strain. Values above zero indicate a positive effect of the treatment relative to control, zero indicates no effect, and negative values indicate a detrimental effect of the pH/alkalinity (or salinity) treatment.

**Figure 6:**
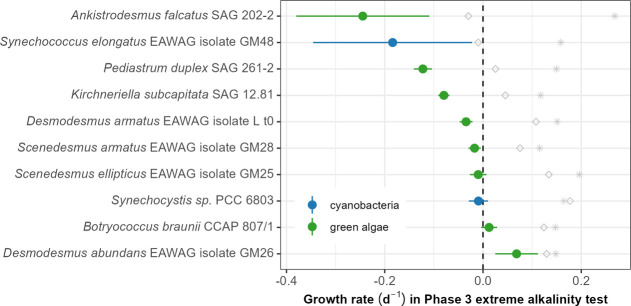
Growth rates of each algae strain in the high-pH and high-alkalinity screening (Phase 3), estimated using the R package “growthTools”. Filled circles and lines represent the mean and 95% confidence interval of growth rate in high pH and alkalinity; gray diamonds show the average growth rates of the salinity treatment (9.5 g/L NaCl); and asterisks show the average growth rates in the control treatment.

### Alkalinity Effects Are Not Driven by Salinity Stress

Increased alkalinity inherently increases salinity in aquatic systems by increasing the concentration of dissolved ionic solutes, creating a link between the two factors that often makes causal and mechanistic inference difficult. We do observe an overall significant relationship between salinity and alkalinity effects on both max. fluorescence (*F*_3,104_ = 68, *p* < 10^–5^) and on growth rate (*F*_2,56_ = 4.2, *p* < 0.01). However, our results across all three phases also show that salinity effects can differ substantially from alkalinity effects; Figure 2A shows the quantitative relationship between the salinity and pH/alkalinity treatment effects on biomass production in all three phases, and that in all phases, the alkalinity–salinity relationship is never one-to-one, as would be expected if salinity stress was driving responses to high-alkalinity treatments. This is further supported by the multiple regression model for effects on biomass concentration, which gives a slope estimate of 0.50 (95% CI: 0.30–0.69), indicating a significant deviation from a 1:1 slope. The relationship between high-salinity growth rates and high-pH/alkalinity growth rates also deviates from the 1:1 line (Figure 2B), but to a lesser extent (slope estimate = 0.70, 95% CI: 0.21–1.2). However, note that the majority of points (66%) lie in quadrant 2 of the figure, i.e., where growth is positive in high salinity but negative in high pH/alkalinity, again indicating clear differences in species responses to the two sets of environmental conditions.

## Discussion

To the best of our knowledge, this study represents the largest and taxonomically broadest screening of algal tolerance to high pH and alkalinity to date and provides much-needed information about the innate ability of freshwater algae to tolerate conditions that are generally considered suitable only for the growth of extremophiles. Our study shows that the magnitude of pH and alkalinity stress significantly alters the suite of species capable of productive growth. The relatively low pH/alkalinity in Phase 1 (pH ∼ 8.5 and 25 mM carbonate alkalinity) yielded growth increases for many algae (including both desired and undesired strains), while increasing the initial pH to 10 and further increasing the alkalinity in Phases 2 and 3 inhibited the majority of strains, again reinforcing the strong selective force of such extreme pH and alkalinity conditions. Additionally, our study begins to disentangle the effects of alkalinity versus salinity, which are generally covarying and, thus, sometimes confounding factors in similar studies. Here, we clearly show that salinity and alkalinity effects are often decoupled across algal groups, as only a small subset of strains appeared to have similar responses to both high-salinity and high-alkalinity growth conditions.

Our study is distinct from previous ones because our high-throughput screening provides relatively broad taxonomic information about tolerance to alkalinity and salinity; however, this broad view prevents an in-depth analysis of each individual strain, as has been the focus of previous studies. Specifically, earlier studies showed how lipid content and other important biochemical characteristics were affected by increased alkalinity, usually with significant increases in lipid accumulation.^43–46^ Table 3 shows the pH and alkalinity levels used as well as the observed growth rate and biomass productivity, in a representative selection of recent studies on algae cultivation with increased alkalinity. In summary, the alkalinity values in this study are intermediate relative to those used in other studies, which range from fairly low alkalinity (<12 mM)^46^ to extremes in which algae grew at up to 1000 mM alkalinity.^47^ The observed growth rates in Phase 2 (up to 1.56 d^–1^ with 75 mM added alkalinity) are in line with growth rates in previous studies (Table 3); however, previous studies show higher growth rates in media similar to our Phase 3 medium, which had 150 mM added alkalinity, indicating greater tolerance for extreme conditions than observed among the strains we tested. For example, *Synechocystis* PCC 6803 did not grow in our 150 mM and pH 10 medium, whereas it did grow in 300 mM alkalinity and pH 9.5 in Chi et al.,^20^ suggesting the importance of relatively small differences in initial pH and other environmental conditions. Note that Table 3 is not meant to be a comprehensive review of the literature; however, a systematic review/meta-analysis of high-alkalinity algae cultivation would indeed be a valuable future contribution.

**Table 3:** Comparison of pH/Alkalinity Levels and Growth Responses in This Study to a Selection of Recently Published Articles Focused on Algae Cultivation under Increased Alkalinity

algae strain	alkalinity (mM)	initial pH	growth rate (d^–1^)	biomass productivity (g/m^2^/d)	references	notes
49 strains	25	8.5	not measured	not measured	Phase 1 of this study	
49 strains	75	10	up to 1.56	not measured	Phase 2 of this study	
10 strains	150	10	up to 0.07	not measured	Phase 3 of this study	
*Nannochloropsis salina*	11.9–23.8	8.4–8.6	up to 0.75	n.r.	White et al.^46^	
*Tetraselmis suecica*	11.9–23.8	8.4–8.6	up to 0.52	n.r.	White et al.^46^	
*Nitzschia* sp.	178.6	n.r.	up to 0.46	n.r.	Chagoya et al.^48^	
*Euhalothece* ZM001	500–1840	9.5–10.8	up to 1.36	up to 1.21 g/L/d	Chi et al.^47^	
*Synechocystis* PCC 6803	10–600	9.5	n.r.	n.r.	Chi et al.^20^	grew at up to 300 mM
*Chlorella sorokiniana* UTEX 1602	10–600	9–9.5	n.r.	n.r.	Chi et al.^20^	grew at up to 100 mM
*Dunaliella* (3 different species)	10–600	8–9	n.r.	n.r.	Chi et al.^20^	grew at up to 600 mM
*Cyanothece* sp.	10–600	8–8.25	n.r.	n.r.	Chi et al.^20^	grew at up to 600 mM
*C. sorokiniana* SLA-04	60	9.9	n.r.	up to 38.5	Vadlamani et al.^18^	
*Cyanobacterium* sp. SSL1	190	10–11.3	up to 4.2	15.2	Gao et al.^23^	
*N. inconspicua* hildebrandi	100–300	9–10	up to 1.44^a^	up to 41.5	Burch et al.^21^	
*Phormidium alkaliphilum*	500	10.5	n.r.	3.1–5.8	Haines et al.^49^	+ microbial consortium
*Arthrospira platensis*	200	9	n.r.	2–14	Jimenez et al.^50^	
*A. platensis*	374	8.5	n.r.	44 mg/L/d	Kolukısaoğlu et al.^51^	
*A. platensis*	200	9.0–9.5	0.024	70 mg/L/d	Jung et al.^52^	
*B. braunii*	11.9	9.0–9.5	n.r.	12 mg/L/d	Zhang et al.^39^	↑ hydrocarbons w/alkalinity

a Estimated from hourly dilution rate; n.r.: not reported by authors in the manuscript.

In contrast to previous studies focusing only on target algae strains, our study clearly shows that the beneficial impacts of moderate added alkalinity (25 mM) on industrial strains could also stimulate the growth of undesired or potentially harmful taxa. Specifically, we observed that the grazer *Poterioochromonas* and the toxic bloom-forming algae *Microcystis* and *Planktothrix* all had increased growth at 25 mM added alkalinity relative to the control (Figures 1 and S1). Therefore, while we know from previous work that moderate increases in alkalinity can serve to increase lipid accumulation, our study suggests that such levels may be insufficient to yield the cobenefit of acting as a crop protection measure. On the other hand, we observed inhibition of all potentially harmful species in our study with higher levels of pH (∼10) and alkalinity (75 mM). Another caveat of our results is that the growth rates we observed would likely differ with different growth conditions (e.g., light, temperature, nutrients, inorganic carbon availability) and that the different initial densities due to the serial transfer experimental design may have introduced some variability in the exact growth rate observed (Figure S7). However, the main outcome of importance is whether a certain strain was able to achieve positive growth in a certain environment, and our results definitively show which strains are (or are not) capable of growth in each high-pH/alkalinity environment. The approach used here is a powerful tool for screening; however, it comes with certain limitations. It is therefore recommended to validate promising cultures and cultivation conditions in larger systems, which allow the possibility of continuously monitoring and controlling pH, alkalinity, inorganic carbon concentrations, and other parameters that microplate screening tests do not readily accommodate.

While our study confirms that extremophiles are indeed rare, it does suggest that the potential of microalgae from relatively “normal” aquatic systems should not be ignored. Previous work has employed bioprospecting efforts and identified productive alkaliphilic algae from alkaline lakes such as Soap Lake in Washington.^19,23^ We suggest that alkaline systems are a clear and logical venue for bioprospecting and that this should continue; however, there may also be hidden potential in your local puddle, as common pond algae like *Scenedesmus* and its relatives, as well as common cyanobacteria, were the most successful in our high-alkalinity screening. A key benefit of bioprospecting for locally sourced alkaliphilic strains is that they are likely to be better adapted to local climate conditions, whereas strains from a select few geographically/climatically distant alkaline lakes may not have traits conferring optimal adaptation to local conditions. Future work may also benefit from combining the high-alkalinity approach with other strategies, e.g., using rationally designed polycultures of alkaline-tolerant strains and probiotic bacteria to stabilize outdoor production, or the use of adaptive laboratory evolution to push the limits of tolerance to high alkalinity and high pH in commercial algae strains. Experimental gradient designs with more treatment levels (i.e., more than the three levels used here) will also further elucidate pH and alkalinity tolerance thresholds to optimize productivity in key algal species. A synergistic approach combining high-pH and high-alkalinity cultivation with other advances in the ecology and engineering of algae farming will likely multiply the observed benefits of such strategies.

## Conclusions

Our study screened 49 algal strains (including green algae, cyanobacteria, diatoms, and others) for their ability to grow in three increasingly harsh levels of pH and alkalinity (as well as equivalent concentrations of added salinity). We show that moderate increases in pH and alkalinity (initial pH values of 8.5 and 25 mM added carbonate alkalinity) cause significant growth increases in many strains; however, these include both desired production strains as well as potentially harmful algae. High alkalinity levels (initial pH of 10 and 75 mM carbonate alkalinity) broadly inhibited growth of most species, with only 9/49 strains showing positive average growth rates and only one species growing in the extreme condition (initial pH of 10 and 150 mM carbonate alkalinity). Salinity increases tended to cause very different growth responses when compared to pH/alkalinity increases, suggesting that the osmotic stress in high-alkalinity media, in most cases, is not as important in driving growth inhibition as pH. This study expands our knowledge of the species pool that we may expect to grow in commercial algae farms using such cultivation strategies and identifies key algal taxa that generally can be highly productive in alkaline cultivation systems at various levels of pH and alkalinity. Alkaline cultivation is a promising strategy for enabling the expansion of outdoor microalgae production, and our study provides a framework that can be used in future bioprospecting efforts to expand the array of alkaline-tolerant algal crops that we can harness to more sustainably feed and fuel society on a changing planet.

## Supplementary Material



## Data Availability

All data and code used are publicly available on Zenodo at https://doi.org/10.5281/zenodo.18625807.

## Notes

Tables with test statistics to assess treatment effects; growth curves for all experimental units; figures analyzing different growth rate estimation methods and initial density effects; and alternative visualizations of figures in the main text (PDF)
