# Supplementary material for: Hunting for Extremophiles: A Systematic Screening of Freshwater Microalgae for Tolerance to High-pH and High-Alkalinity Cultivation
Source: ACS Sustain Chem Eng. 2026 Feb 24;14(9):4376–86. doi: 10.1021/acssuschemeng.5c09906 (PMC12977154; doi:10.1021/acssuschemeng.5c09906)
Supplement: Supplementary file 1 [file sc5c09906_si_001.pdf]

Supporting information for:

Hunting for extremophiles: a systematic screening of freshwater microalgae for tolerance to high pH and high alkalinity cultivation

Patrick K. Thomas[1,2*], Robin Gerlach[2,3], Anita Narwani[1]

[1]Department of Aquatic Ecology, Swiss Federal Institute of Aquatic Science and Technology (EAWAG), Dübendorf, Switzerland

[2]Center for Biofilm Engineering, Montana State University, Bozeman, MT, USA

[3]Department of Chemical and Biological Engineering, Montana State University, Bozeman, MT, USA

*Current address of corresponding author: Patrick Thomas, Center for Biofilm Engineering, Montana State University, Bozeman, MT, USA. Email: patrick.thomas4@montana.edu

ORCID IDs - PT: 0000-0002-7259-5766; RG: 0000-0002-7669-3072; AN: 0000-0003-4561-0163

Contents (23 pages):

A. Supplementary Tables

B. Supplementary Figures

A. **Supplementary Tables**

Table S1. Post hoc Games Howell test results showing differences in maximum fluorescence between treatments in Phase 1. "estimate" refers to the estimated mean difference of the first treatment versus the second treatment listed in the "contrast" column (i.e., the negative estimate in the first row means the pH & alkalinity treatment had lower max. fluorescence than the control). "conf.low" and "conf.high" show bounds of 95% confidence intervals; "p.adj" shows adjusted p values, and "p.adj.signif" shows the significance level of the contrast (ns: p>0.05, *: p<0.05; **p<0.01, ***p<0.001, ****p<0.0001).

species	strain	contrast	estimate	conf.low	conf.high	p.adj	p.adj.signif
Anabaena planctonica	NIVA-CYA 651	pH & Alkalinity vs Control	-0.0762	-0.1523	-2.00E-04	0.05	*
Anabaena planctonica	NIVA-CYA 651	Salinity vs Control	0.8208	0.7444	0.8973	0	****
Anabaena planctonica	NIVA-CYA 651	Salinity vs pH & Alkalinity	0.8971	0.8323	0.9618	0	****
Ankistrodesmus falcatus	SAG 202-2	pH & Alkalinity vs Control	0.2376	0.121	0.3542	0.002	**
Ankistrodesmus falcatus	SAG 202-2	Salinity vs Control	-0.2011	-0.3195	-0.0827	0.01	**
Ankistrodesmus falcatus	SAG 202-2	Salinity vs pH & Alkalinity	-0.4387	-0.5218	-0.3556	1.00E-04	***
Aphanizomenon flos-aquae	NIVA-CYA 681	pH & Alkalinity vs Control	0.0026	-0.0394	0.0446	0.979	ns
Aphanizomenon flos-aquae	NIVA-CYA 681	Salinity vs Control	0.0044	-0.0421	0.0509	0.955	ns
Aphanizomenon flos-aquae	NIVA-CYA 681	Salinity vs pH & Alkalinity	0.0017	-0.0408	0.0443	0.991	ns
Botryococcus braunii	CCAP 807/1	pH & Alkalinity vs Control	0.1642	0.0663	0.262	0.005	**
Botryococcus braunii	CCAP 807/1	Salinity vs Control	0.0046	-0.2104	0.2195	0.997	ns
Botryococcus braunii	CCAP 807/1	Salinity vs pH & Alkalinity	-0.1596	-0.3744	0.0552	0.117	ns
Botryococcus braunii	CCAP 807/2	pH & Alkalinity vs Control	-0.0791	-0.1851	0.0269	0.106	ns
Botryococcus braunii	CCAP 807/2	Salinity vs Control	-0.1175	-0.2083	-0.0266	0.024	*
Botryococcus braunii	CCAP 807/2	Salinity vs pH & Alkalinity	-0.0384	-0.1444	0.0675	0.539	ns
Botryococcus braunii	EAWAG isolate PT1	pH & Alkalinity vs Control	-0.4223	-0.6865	-0.1581	0.008	**
Botryococcus braunii	EAWAG isolate PT1	Salinity vs Control	-0.3066	-0.4748	-0.1385	0.004	**
Botryococcus braunii	EAWAG isolate PT1	Salinity vs pH & Alkalinity	0.1157	-0.1494	0.3808	0.366	ns
Botryococcus braunii	NIES 2199	pH & Alkalinity vs Control	0.0568	-0.0106	0.1241	0.09	ns
Botryococcus braunii	NIES 2199	Salinity vs Control	-0.0655	-0.1195	-0.0114	0.023	*
Botryococcus braunii	NIES 2199	Salinity vs pH & Alkalinity	-0.1222	-0.1876	-0.0568	0.004	**

Botryococcus braunii	SAG 30.81	pH & Alkalinity vs Control	0.018	-0.0686	0.1046	0.796	ns
Botryococcus braunii	SAG 30.81	Salinity vs Control	-0.1787	-0.2693	-0.0881	0.002	**
Botryococcus braunii	SAG 30.81	Salinity vs pH & Alkalinity	-0.1967	-0.2735	-0.1199	6.00E-04	***
Botryococcus braunii	Showa	pH & Alkalinity vs Control	-0.7966	-1.0886	-0.5046	4.00E-04	***
Botryococcus braunii	Showa	Salinity vs Control	-0.1987	-0.4547	0.0573	0.107	ns
Botryococcus braunii	Showa	Salinity vs pH & Alkalinity	0.5979	0.3389	0.8569	0.002	**
Chlamydomonas reinhardtii	CC 1690	pH & Alkalinity vs Control	-0.0314	-0.2042	0.1414	0.767	ns
Chlamydomonas reinhardtii	CC 1690	Salinity vs Control	-0.0981	-0.2699	0.0738	0.196	ns
Chlamydomonas reinhardtii	CC 1690	Salinity vs pH & Alkalinity	-0.0667	-0.1056	-0.0277	0.005	**
Chlamydomonas reinhardtii	CC 3060	pH & Alkalinity vs Control	0.7855	0.5522	1.0188	0.001	***
Chlamydomonas reinhardtii	CC 3060	Salinity vs Control	-0.0057	-0.2408	0.2294	0.995	ns
Chlamydomonas reinhardtii	CC 3060	Salinity vs pH & Alkalinity	-0.7912	-0.8468	-0.7357	0	****
Chlorella vulgaris	SAG 211-11b	pH & Alkalinity vs Control	0.3821	0.1924	0.5718	0.007	**
Chlorella vulgaris	SAG 211-11b	Salinity vs Control	-0.1143	-0.1333	-0.0952	0	****
Chlorella vulgaris	SAG 211-11b	Salinity vs pH & Alkalinity	-0.4964	-0.6869	-0.3058	0.003	**
Cosmarium botrytis	SAG 136.8	pH & Alkalinity vs Control	0.1384	-0.3311	0.608	0.657	ns
Cosmarium botrytis	SAG 136.8	Salinity vs Control	-0.2452	-0.6568	0.1664	0.212	ns
Cosmarium botrytis	SAG 136.8	Salinity vs pH & Alkalinity	-0.3836	-0.7973	0.0301	0.064	ns
Cryptomonas sp.	NIVA 3/09	pH & Alkalinity vs Control	-0.4894	-0.6057	-0.3731	0	****
Cryptomonas sp.	NIVA 3/09	Salinity vs Control	-0.4106	-0.8361	0.015	0.055	ns
Cryptomonas sp.	NIVA 3/09	Salinity vs pH & Alkalinity	0.0789	-0.3528	0.5105	0.768	ns
Cyclotella meneghiniana	SAG 1020-1a	pH & Alkalinity vs Control	1.0163	0.9194	1.1133	0	****
Cyclotella meneghiniana	SAG 1020-1a	Salinity vs Control	0.6915	0.58	0.803	0	****
Cyclotella meneghiniana	SAG 1020-1a	Salinity vs pH & Alkalinity	-0.3249	-0.4237	-0.226	4.00E-04	***
Cystodinium sp.	SAG 59.87	pH & Alkalinity vs Control	-0.87	-1.1162	-0.6238	5.00E-04	***
Cystodinium sp.	SAG 59.87	Salinity vs Control	-0.4196	-0.8534	0.0142	0.056	ns
Cystodinium sp.	SAG 59.87	Salinity vs pH & Alkalinity	0.4504	-0.0076	0.9084	0.052	ns
Desmodesmus abundans	EAWAG isolate GM26	pH & Alkalinity vs Control	-0.1968	-0.2693	-0.1244	0.001	***
Desmodesmus abundans	EAWAG isolate GM26	Salinity vs Control	-0.3423	-0.4155	-0.269	0	****

Desmodesmus abundans	EAWAG isolate GM26	Salinity vs pH & Alkalinity	-0.1454	-0.1959	-0.095	4.00E-04	***
Desmodesmus armatus	EAWAG isolate L t0	pH & Alkalinity vs Control	0.3685	0.3301	0.407	0	****
Desmodesmus armatus	EAWAG isolate L t0	Salinity vs Control	-0.2052	-0.249	-0.1614	0	****
Desmodesmus armatus	EAWAG isolate L t0	Salinity vs pH & Alkalinity	-0.5738	-0.6182	-0.5293	0	****
Fistulifera saprophila	EAWAG isolate GM16	pH & Alkalinity vs Control	1.3991	0.0818	2.7163	0.042	*
Fistulifera saprophila	EAWAG isolate GM16	Salinity vs Control	0.0632	-1.2131	1.3395	0.983	ns
Fistulifera saprophila	EAWAG isolate GM16	Salinity vs pH & Alkalinity	-1.3358	-1.8805	-0.7912	0.001	***
Fragilaria capucina	CCAC 2678B	pH & Alkalinity vs Control	-0.0793	-0.3438	0.1852	0.557	ns
Fragilaria capucina	CCAC 2678B	Salinity vs Control	-0.0691	-0.329	0.1908	0.692	ns
Fragilaria capucina	CCAC 2678B	Salinity vs pH & Alkalinity	0.0102	-0.1656	0.186	0.978	ns
Fragilaria crotonensis	EAWAG isolate GM2	pH & Alkalinity vs Control	-0.042	-0.8779	0.7938	0.977	ns
Fragilaria crotonensis	EAWAG isolate GM2	Salinity vs Control	-0.1313	-0.9689	0.7064	0.81	ns
Fragilaria crotonensis	EAWAG isolate GM2	Salinity vs pH & Alkalinity	-0.0892	-0.2126	0.0342	0.146	ns
Fragilaria mesolepta	EAWAG isolate GM15	pH & Alkalinity vs Control	-0.8985	-1.0699	-0.727	0	****
Fragilaria mesolepta	EAWAG isolate GM15	Salinity vs Control	-0.9421	-1.0981	-0.7862	1.00E-04	****
Fragilaria mesolepta	EAWAG isolate GM15	Salinity vs pH & Alkalinity	-0.0436	-0.1913	0.104	0.594	ns
Kirchneriella subcapitata	SAG 12.81	pH & Alkalinity vs Control	0.4008	0.3304	0.4713	1.00E-04	***
Kirchneriella subcapitata	SAG 12.81	Salinity vs Control	-0.1553	-0.1881	-0.1225	0	****
Kirchneriella subcapitata	SAG 12.81	Salinity vs pH & Alkalinity	-0.5561	-0.6251	-0.4872	0	****
Lagerheimia hindakii	SAG 11.92	pH & Alkalinity vs Control	0.1732	0.1254	0.221	1.00E-04	***
Lagerheimia hindakii	SAG 11.92	Salinity vs Control	-0.1808	-0.4989	0.1374	0.19	ns
Lagerheimia hindakii	SAG 11.92	Salinity vs pH & Alkalinity	-0.354	-0.6697	-0.0383	0.037	*
Lagerheimia subsalsa	EAWAG isolate GM37	pH & Alkalinity vs Control	0.4609	0.0437	0.8782	0.038	*
Lagerheimia subsalsa	EAWAG isolate GM37	Salinity vs Control	-0.5407	-0.9463	-0.1351	0.02	*
Lagerheimia subsalsa	EAWAG isolate GM37	Salinity vs pH & Alkalinity	-1.0016	-1.1556	-0.8477	0	****
Medakamo hakoo	NIES 4000	pH & Alkalinity vs Control	0.3372	0.2832	0.3913	0	****
Medakamo hakoo	NIES 4000	Salinity vs Control	-0.0349	-0.0892	0.0194	0.188	ns
Medakamo hakoo	NIES 4000	Salinity vs pH & Alkalinity	-0.3721	-0.4078	-0.3364	0	****
Messastrum gracile	EAWAG isolate GM45	pH & Alkalinity vs Control	-0.0035	-0.1667	0.1596	0.997	ns

Messastrum gracile	EAWAG isolate GM45	Salinity vs Control	-0.0122	-0.1796	0.1552	0.961	ns
Messastrum gracile	EAWAG isolate GM45	Salinity vs pH & Alkalinity	-0.0087	-0.0883	0.0709	0.937	ns
Micractinium pusillum	CCAP 231/1	pH & Alkalinity vs Control	-0.0923	-0.14	-0.0446	0.006	**
Micractinium pusillum	CCAP 231/1	Salinity vs Control	0.0373	-0.007	0.0815	0.081	ns
Micractinium pusillum	CCAP 231/1	Salinity vs pH & Alkalinity	0.1296	0.0787	0.1805	6.00E-04	***
Microcystis aeruginosa	EAWAG isolate Greifensee	pH & Alkalinity vs Control	-0.9326	-1.0146	-0.8507	0	****
Microcystis aeruginosa	EAWAG isolate Greifensee	Salinity vs Control	-0.7785	-0.8166	-0.7403	0	****
Microcystis aeruginosa	EAWAG isolate Greifensee	Salinity vs pH & Alkalinity	0.1542	0.077	0.2313	0.004	**
Microcystis aeruginosa	PCC 7806	pH & Alkalinity vs Control	0.3855	0.2948	0.4762	1.00E-04	****
Microcystis aeruginosa	PCC 7806	Salinity vs Control	0.3133	0.03	0.5967	0.038	*
Microcystis aeruginosa	PCC 7806	Salinity vs pH & Alkalinity	-0.0722	-0.3488	0.2045	0.633	ns
Nannochloropsis limnetica	SAG 18.99	pH & Alkalinity vs Control	0.1276	0.0195	0.2357	0.031	*
Nannochloropsis limnetica	SAG 18.99	Salinity vs Control	-0.1232	-0.1599	-0.0865	1.00E-04	***
Nannochloropsis limnetica	SAG 18.99	Salinity vs pH & Alkalinity	-0.2508	-0.3575	-0.144	0.003	**
Nannochloropsis oculata	SAG 38.85	pH & Alkalinity vs Control	0.6719	0.5984	0.7454	0	****
Nannochloropsis oculata	SAG 38.85	Salinity vs Control	0.7178	0.6461	0.7896	0	****
Nannochloropsis oculata	SAG 38.85	Salinity vs pH & Alkalinity	0.0459	-0.0364	0.1282	0.276	ns
Oocystella heteromucosa	EAWAG isolate GM35	pH & Alkalinity vs Control	0.2114	-0.1338	0.5566	0.165	ns
Oocystella heteromucosa	EAWAG isolate GM35	Salinity vs Control	-0.474	-0.734	-0.2139	0.009	**
Oocystella heteromucosa	EAWAG isolate GM35	Salinity vs pH & Alkalinity	-0.6854	-1.0164	-0.3544	0.002	**
Oocystis solitaria	SAG 83.8	pH & Alkalinity vs Control	0.101	-0.0522	0.2543	0.183	ns
Oocystis solitaria	SAG 83.8	Salinity vs Control	-0.0115	-0.1461	0.1231	0.963	ns
Oocystis solitaria	SAG 83.8	Salinity vs pH & Alkalinity	-0.1125	-0.2696	0.0445	0.148	ns
Oocystis sp.	EAWAG isolate L t0	pH & Alkalinity vs Control	0.3866	0.3017	0.4714	0	****
Oocystis sp.	EAWAG isolate L t0	Salinity vs Control	-0.1716	-0.2556	-0.0876	0.002	**
Oocystis sp.	EAWAG isolate L t0	Salinity vs pH & Alkalinity	-0.5581	-0.6369	-0.4794	0	****
Pandorina unicocca	EAWAG isolate GM17	pH & Alkalinity vs Control	-1.115	-1.2154	-1.0145	0	****
Pandorina unicocca	EAWAG isolate GM17	Salinity vs Control	-1.2109	-1.354	-1.0678	0	****
Pandorina unicocca	EAWAG isolate GM17	Salinity vs pH & Alkalinity	-0.0959	-0.2398	0.0479	0.171	ns

Pediastrum boryanum	EAWAG isolate GM20	pH & Alkalinity vs Control	0.833	0.4135	1.2525	0.006	**
Pediastrum boryanum	EAWAG isolate GM20	Salinity vs Control	0.3672	0.2661	0.4683	1.00E-04	***
Pediastrum boryanum	EAWAG isolate GM20	Salinity vs pH & Alkalinity	-0.4658	-0.8911	-0.0405	0.039	*
Pediastrum boryanum	SAG 87.81	pH & Alkalinity vs Control	-0.2798	-0.426	-0.1337	0.005	**
Pediastrum boryanum	SAG 87.81	Salinity vs Control	-0.2473	-0.3942	-0.1005	0.009	**
Pediastrum boryanum	SAG 87.81	Salinity vs pH & Alkalinity	0.0325	-0.0312	0.0962	0.329	ns
Pediastrum duplex	EAWAG isolate GM22	pH & Alkalinity vs Control	-0.7912	-0.9068	-0.6756	0	****
Pediastrum duplex	EAWAG isolate GM22	Salinity vs Control	-0.7798	-0.9742	-0.5853	1.00E-04	***
Pediastrum duplex	EAWAG isolate GM22	Salinity vs pH & Alkalinity	0.0114	-0.1857	0.2085	0.977	ns
Pediastrum duplex	SAG 261-2	pH & Alkalinity vs Control	0.5113	0.1585	0.864	0.018	*
Pediastrum duplex	SAG 261-2	Salinity vs Control	-0.0759	-0.2269	0.0751	0.246	ns
Pediastrum duplex	SAG 261-2	Salinity vs pH & Alkalinity	-0.5872	-0.9131	-0.2613	0.006	**
Planktothrix rubescens	NIVA-CYA 619	pH & Alkalinity vs Control	0.3342	-0.0838	0.7522	0.088	ns
Planktothrix rubescens	NIVA-CYA 619	Salinity vs Control	0.0477	-0.3726	0.4679	0.889	ns
Planktothrix rubescens	NIVA-CYA 619	Salinity vs pH & Alkalinity	-0.2865	-0.324	-0.249	0	****
Poterioochromonas malhamensis	CCAP 933/1C	pH & Alkalinity vs Control	0.5737	-0.0141	1.1615	0.055	ns
Poterioochromonas malhamensis	CCAP 933/1C	Salinity vs Control	0.487	-0.0325	1.0065	0.06	ns
Poterioochromonas malhamensis	CCAP 933/1C	Salinity vs pH & Alkalinity	-0.0867	-0.6456	0.4722	0.829	ns
Scenedesmus acuminatus	SAG 38.81	pH & Alkalinity vs Control	-0.0243	-0.0864	0.0379	0.474	ns
Scenedesmus acuminatus	SAG 38.81	Salinity vs Control	-0.1563	-0.247	-0.0655	0.007	**
Scenedesmus acuminatus	SAG 38.81	Salinity vs pH & Alkalinity	-0.132	-0.2234	-0.0406	0.012	*
Scenedesmus armatus	EAWAG isolate GM28	pH & Alkalinity vs Control	0.3769	0.2557	0.4981	5.00E-04	***
Scenedesmus armatus	EAWAG isolate GM28	Salinity vs Control	-0.2465	-0.3164	-0.1765	1.00E-04	***
Scenedesmus armatus	EAWAG isolate GM28	Salinity vs pH & Alkalinity	-0.6234	-0.7452	-0.5015	1.00E-04	****
Scenedesmus ellipticus	EAWAG isolate GM25	pH & Alkalinity vs Control	0.2926	0.2155	0.3697	5.00E-04	***
Scenedesmus ellipticus	EAWAG isolate GM25	Salinity vs Control	-0.3619	-0.442	-0.2818	0	****
Scenedesmus ellipticus	EAWAG isolate GM25	Salinity vs pH & Alkalinity	-0.6545	-0.7194	-0.5896	0	****
Staurastrum punctulatum	SAG 679-1	pH & Alkalinity vs Control	-0.7828	-0.9325	-0.6332	0	****
Staurastrum punctulatum	SAG 679-1	Salinity vs Control	-0.7295	-1.0042	-0.4547	0.001	***

Staurastrum punctulatum	SAG 679-1	Salinity vs pH & Alkalinity	0.0534	-0.227	0.3337	0.78	ns
Synechococcus elongatus	EAWAG isolate GM48	pH & Alkalinity vs Control	0.1115	0.0629	0.1601	0.004	**
Synechococcus elongatus	EAWAG isolate GM48	Salinity vs Control	0.2635	0.2146	0.3124	0	****
Synechococcus elongatus	EAWAG isolate GM48	Salinity vs pH & Alkalinity	0.152	0.1116	0.1924	7.00E-04	***
Synechocystis sp.	PCC 6803	pH & Alkalinity vs Control	0.2459	0.003	0.4889	0.048	*
Synechocystis sp.	PCC 6803	Salinity vs Control	0.0222	-0.2115	0.2559	0.93	ns
Synechocystis sp.	PCC 6803	Salinity vs pH & Alkalinity	-0.2237	-0.2836	-0.1638	7.00E-04	***
Tetraedron minimum	SAG 44.81	pH & Alkalinity vs Control	0.025	-0.2801	0.3301	0.94	ns
Tetraedron minimum	SAG 44.81	Salinity vs Control	-0.3152	-0.6102	-0.0203	0.041	*
Tetraedron minimum	SAG 44.81	Salinity vs pH & Alkalinity	-0.3402	-0.4108	-0.2696	2.00E-04	***

Table S2. Post hoc Games Howell test results showing differences in maximum fluorescence between treatments in Phase 2. “estimate” refers to the estimated mean difference of the first treatment versus the second treatment listed in the “contrast” column (i.e., the negative estimate in the first row means the pH & alkalinity treatment had lower max. fluorescence than the control). “conf.low” and “conf.high” show bounds of 95% confidence intervals; “p.adj” shows adjusted p values, and “p.adj.signif” shows the significance level of the contrast (ns: p>0.05, *: p<0.05; **p<0.01, ***p<0.001, ****p<0.0001).

species	strain	contrast	estimate	conf.low	conf.high	p.adj	p.adj.signif
Anabaena planctonica	NIVA-CYA 651	pH & Alkalinity vs Control	-4.3147	-5.2263	-3.4031	3.00E-04	***
Anabaena planctonica	NIVA-CYA 651	Salinity vs Control	-2.9382	-7.0454	1.1689	0.119	ns
Anabaena planctonica	NIVA-CYA 651	Salinity vs pH & Alkalinity	1.3765	-2.8612	5.6141	0.464	ns
Ankistrodesmus falcatus	SAG 202-2	pH & Alkalinity vs Control	-1.7618	-2.0567	-1.4669	1.00E-04	****
Ankistrodesmus falcatus	SAG 202-2	Salinity vs Control	-0.7747	-0.9161	-0.6333	0	****
Ankistrodesmus falcatus	SAG 202-2	Salinity vs pH & Alkalinity	0.9871	0.6942	1.28	5.00E-04	***
Aphanizomenon flos-aquae	NIVA-CYA 681	pH & Alkalinity vs Control	-4.7031	-5.0156	-4.3907	0	****
Aphanizomenon flos-aquae	NIVA-CYA 681	Salinity vs Control	-1.6606	-2.4125	-0.9086	0.003	**
Aphanizomenon flos-aquae	NIVA-CYA 681	Salinity vs pH & Alkalinity	3.0426	2.2457	3.8395	9.00E-04	***
Botryococcus braunii	CCAP 807/1	pH & Alkalinity vs Control	-1.8172	-2.2813	-1.3532	8.00E-04	***
Botryococcus braunii	CCAP 807/1	Salinity vs Control	-0.0582	-0.2659	0.1494	0.62	ns
Botryococcus braunii	CCAP 807/1	Salinity vs pH & Alkalinity	1.759	1.3213	2.1967	2.00E-04	***
Botryococcus braunii	CCAP 807/2	pH & Alkalinity vs Control	-1.253	-1.4533	-1.0527	0	****
Botryococcus braunii	CCAP 807/2	Salinity vs Control	-0.2653	-0.4324	-0.0982	0.007	**
Botryococcus braunii	CCAP 807/2	Salinity vs pH & Alkalinity	0.9878	0.801	1.1745	0	****
Botryococcus braunii	EAWAG isolate PT1	pH & Alkalinity vs Control	-2.9165	-3.3135	-2.5195	0	****
Botryococcus braunii	EAWAG isolate PT1	Salinity vs Control	-0.9922	-1.2786	-0.7057	1.00E-04	***
Botryococcus braunii	EAWAG isolate PT1	Salinity vs pH & Alkalinity	1.9243	1.5217	2.3269	0	****
Botryococcus braunii	NIES 2199	pH & Alkalinity vs Control	-2.2304	-2.3376	-2.1232	0	****
Botryococcus braunii	NIES 2199	Salinity vs Control	-0.4489	-0.574	-0.3237	1.00E-04	***
Botryococcus braunii	NIES 2199	Salinity vs pH & Alkalinity	1.7816	1.6577	1.9054	0	****
Botryococcus braunii	SAG 30.81	pH & Alkalinity vs Control	-0.3984	-0.6909	-0.1058	0.017	*
Botryococcus braunii	SAG 30.81	Salinity vs Control	-0.1318	-0.3203	0.0567	0.157	ns

Botryococcus braunii	SAG 30.81	Salinity vs pH & Alkalinity	0.2666	-0.0262	0.5594	0.069	ns
Botryococcus braunii	Showa	pH & Alkalinity vs Control	-3.4367	-4.1691	-2.7043	5.00E-04	***
Botryococcus braunii	Showa	Salinity vs Control	-1.6073	-2.29	-0.9246	0.002	**
Botryococcus braunii	Showa	Salinity vs pH & Alkalinity	1.8294	1.4218	2.237	4.00E-04	***
Chlamydomonas reinhardtii	CC 1690	pH & Alkalinity vs Control	-2.8908	-3.0969	-2.6846	0	****
Chlamydomonas reinhardtii	CC 1690	Salinity vs Control	-0.8191	-0.9043	-0.7338	0	****
Chlamydomonas reinhardtii	CC 1690	Salinity vs pH & Alkalinity	2.0717	1.8641	2.2793	0	****
Chlamydomonas reinhardtii	CC 3060	pH & Alkalinity vs Control	-4.4377	-6.0459	-2.8295	0.003	**
Chlamydomonas reinhardtii	CC 3060	Salinity vs Control	-0.6351	-0.9136	-0.3565	0.001	***
Chlamydomonas reinhardtii	CC 3060	Salinity vs pH & Alkalinity	3.8026	2.2125	5.3927	0.004	**
Chlorella vulgaris	SAG 211-11b	pH & Alkalinity vs Control	-0.9304	-1.3047	-0.5561	0.004	**
Chlorella vulgaris	SAG 211-11b	Salinity vs Control	0.0094	-0.1157	0.1346	0.952	ns
Chlorella vulgaris	SAG 211-11b	Salinity vs pH & Alkalinity	0.9399	0.5879	1.2918	0.002	**
Cosmarium botrytis	SAG 136.8	pH & Alkalinity vs Control	-4.0541	-4.7918	-3.3164	1.00E-04	****
Cosmarium botrytis	SAG 136.8	Salinity vs Control	-2.5819	-2.9894	-2.1744	0	****
Cosmarium botrytis	SAG 136.8	Salinity vs pH & Alkalinity	1.4721	0.7335	2.2108	0.004	**
Cryptomonas sp.	NIVA 3/09	pH & Alkalinity vs Control	-2.5528	-4.785	-0.3206	0.035	*
Cryptomonas sp.	NIVA 3/09	Salinity vs Control	-2.2383	-4.3596	-0.117	0.042	*
Cryptomonas sp.	NIVA 3/09	Salinity vs pH & Alkalinity	0.3145	-0.499	1.1281	0.445	ns
Cyclotella meneghiniana	SAG 1020-1a	pH & Alkalinity vs Control	-2.5442	-2.8304	-2.258	0	****
Cyclotella meneghiniana	SAG 1020-1a	Salinity vs Control	-0.0247	-0.2963	0.2469	0.938	ns
Cyclotella meneghiniana	SAG 1020-1a	Salinity vs pH & Alkalinity	2.5195	2.4338	2.6052	0	****
Cystodinium sp.	SAG 59.87	pH & Alkalinity vs Control	-1.6612	-2.2966	-1.0258	0.002	**
Cystodinium sp.	SAG 59.87	Salinity vs Control	-0.8278	-1.3722	-0.2834	0.012	*
Cystodinium sp.	SAG 59.87	Salinity vs pH & Alkalinity	0.8334	0.1629	1.5039	0.021	*
Desmodesmus abundans	EAWAG isolate GM26	pH & Alkalinity vs Control	-0.1615	-0.3149	-0.0082	0.042	*
Desmodesmus abundans	EAWAG isolate GM26	Salinity vs Control	-0.2109	-0.3019	-0.1198	0.001	***
Desmodesmus abundans	EAWAG isolate GM26	Salinity vs pH & Alkalinity	-0.0493	-0.2026	0.1039	0.565	ns
Desmodesmus armatus	EAWAG isolate L t0	pH & Alkalinity vs Control	-0.6657	-0.7379	-0.5934	0	****

Desmodesmus armatus	EAWAG isolate L t0	Salinity vs Control	-0.2871	-0.3126	-0.2616	0	****
Desmodesmus armatus	EAWAG isolate L t0	Salinity vs pH & Alkalinity	0.3786	0.3087	0.4484	1.00E-04	***
Fistulifera saprophila	EAWAG isolate GM16	pH & Alkalinity vs Control	-4.6057	-8.0122	-1.1991	0.022	*
Fistulifera saprophila	EAWAG isolate GM16	Salinity vs Control	-0.763	-2.8318	1.3058	0.395	ns
Fistulifera saprophila	EAWAG isolate GM16	Salinity vs pH & Alkalinity	3.8427	0.7208	6.9647	0.023	*
Fragilaria capucina	CCAC 2678B	pH & Alkalinity vs Control	-3.0175	-6.5177	0.4828	0.073	ns
Fragilaria capucina	CCAC 2678B	Salinity vs Control	-3.1149	-6.609	0.3792	0.067	ns
Fragilaria capucina	CCAC 2678B	Salinity vs pH & Alkalinity	-0.0974	-0.3873	0.1924	0.581	ns
Fragilaria crotonensis	EAWAG isolate GM2	pH & Alkalinity vs Control	-2.4309	-2.8544	-2.0074	1.00E-04	****
Fragilaria crotonensis	EAWAG isolate GM2	Salinity vs Control	-2.3659	-2.5532	-2.1786	0	****
Fragilaria crotonensis	EAWAG isolate GM2	Salinity vs pH & Alkalinity	0.065	-0.3667	0.4967	0.848	ns
Fragilaria mesolepta	EAWAG isolate GM15	pH & Alkalinity vs Control	-4.2354	-4.5297	-3.9411	0	****
Fragilaria mesolepta	EAWAG isolate GM15	Salinity vs Control	-4.4203	-4.7665	-4.0742	0	****
Fragilaria mesolepta	EAWAG isolate GM15	Salinity vs pH & Alkalinity	-0.1849	-0.5406	0.1707	0.315	ns
Kirchneriella subcapitata	SAG 12.81	pH & Alkalinity vs Control	-1.3731	-1.439	-1.3071	0	****
Kirchneriella subcapitata	SAG 12.81	Salinity vs Control	-0.1572	-0.2175	-0.0969	0.001	***
Kirchneriella subcapitata	SAG 12.81	Salinity vs pH & Alkalinity	1.2159	1.1438	1.2879	0	****
Lagerheimia hindakii	SAG 11.92	pH & Alkalinity vs Control	-1.4273	-1.5084	-1.3461	0	****
Lagerheimia hindakii	SAG 11.92	Salinity vs Control	-0.2461	-0.3513	-0.1409	0.001	***
Lagerheimia hindakii	SAG 11.92	Salinity vs pH & Alkalinity	1.1811	1.0787	1.2836	0	****
Lagerheimia subsalsa	EAWAG isolate GM37	pH & Alkalinity vs Control	-1.2853	-1.7033	-0.8673	0.002	**
Lagerheimia subsalsa	EAWAG isolate GM37	Salinity vs Control	-0.7271	-1.1189	-0.3352	0.006	**
Lagerheimia subsalsa	EAWAG isolate GM37	Salinity vs pH & Alkalinity	0.5583	0.3755	0.741	0.001	***
Medakamo hakoo	NIES 4000	pH & Alkalinity vs Control	-1.2016	-1.2855	-1.1177	0	****
Medakamo hakoo	NIES 4000	Salinity vs Control	-0.2274	-0.2938	-0.1611	1.00E-04	***
Medakamo hakoo	NIES 4000	Salinity vs pH & Alkalinity	0.9742	0.8939	1.0544	0	****
Messastrum gracile	EAWAG isolate GM45	pH & Alkalinity vs Control	-1.8183	-2.126	-1.5106	1.00E-04	****
Messastrum gracile	EAWAG isolate GM45	Salinity vs Control	0.0713	-0.0467	0.1892	0.204	ns
Messastrum gracile	EAWAG isolate GM45	Salinity vs pH & Alkalinity	1.8895	1.5667	2.2124	2.00E-04	***

Micractinium pusillum	CCAP 231/1	pH & Alkalinity vs Control	-2.5993	-3.0135	-2.1851	1.00E-04	***
Micractinium pusillum	CCAP 231/1	Salinity vs Control	-0.1548	-0.264	-0.0455	0.012	*
Micractinium pusillum	CCAP 231/1	Salinity vs pH & Alkalinity	2.4445	2.0221	2.8669	3.00E-04	***
Microcystis aeruginosa	EAWAG isolate Greifensee	pH & Alkalinity vs Control	-4.1129	-4.2129	-4.013	0	****
Microcystis aeruginosa	EAWAG isolate Greifensee	Salinity vs Control	-1.0917	-1.277	-0.9064	0	****
Microcystis aeruginosa	EAWAG isolate Greifensee	Salinity vs pH & Alkalinity	3.0212	2.8305	3.212	0	****
Microcystis aeruginosa	PCC 7806	pH & Alkalinity vs Control	-2.9838	-3.2555	-2.7121	0	****
Microcystis aeruginosa	PCC 7806	Salinity vs Control	-0.3679	-0.4492	-0.2866	0	****
Microcystis aeruginosa	PCC 7806	Salinity vs pH & Alkalinity	2.616	2.3461	2.8859	0	****
Nannochloropsis limnetica	SAG 18.99	pH & Alkalinity vs Control	-2.5367	-2.6534	-2.42	0	****
Nannochloropsis limnetica	SAG 18.99	Salinity vs Control	-1.3775	-1.4774	-1.2776	0	****
Nannochloropsis limnetica	SAG 18.99	Salinity vs pH & Alkalinity	1.1591	1.0526	1.2657	0	****
Nannochloropsis oculata	SAG 38.85	pH & Alkalinity vs Control	-1.9518	-2.0434	-1.8603	0	****
Nannochloropsis oculata	SAG 38.85	Salinity vs Control	0.2296	0.1848	0.2745	2.00E-04	***
Nannochloropsis oculata	SAG 38.85	Salinity vs pH & Alkalinity	2.1814	2.0835	2.2794	0	****
Oocystella heteromucosa	EAWAG isolate GM35	pH & Alkalinity vs Control	-2.6559	-3.0435	-2.2683	1.00E-04	***
Oocystella heteromucosa	EAWAG isolate GM35	Salinity vs Control	-2.8654	-3.19	-2.5409	0	****
Oocystella heteromucosa	EAWAG isolate GM35	Salinity vs pH & Alkalinity	-0.2096	-0.5876	0.1685	0.277	ns
Oocystis solitaria	SAG 83.8	pH & Alkalinity vs Control	-2.7447	-3.0771	-2.4122	0	****
Oocystis solitaria	SAG 83.8	Salinity vs Control	-1.5293	-1.7442	-1.3144	1.00E-04	****
Oocystis solitaria	SAG 83.8	Salinity vs pH & Alkalinity	1.2154	0.8956	1.5351	1.00E-04	***
Oocystis sp.	EAWAG isolate L t0	pH & Alkalinity vs Control	-2.2561	-2.555	-1.9572	1.00E-04	****
Oocystis sp.	EAWAG isolate L t0	Salinity vs Control	-0.2899	-0.3465	-0.2333	0	****
Oocystis sp.	EAWAG isolate L t0	Salinity vs pH & Alkalinity	1.9662	1.6661	2.2663	2.00E-04	***
Pandorina unicocca	EAWAG isolate GM17	pH & Alkalinity vs Control	-3.918	-4.0196	-3.8164	0	****
Pandorina unicocca	EAWAG isolate GM17	Salinity vs Control	-3.7789	-4.1808	-3.377	0	****
Pandorina unicocca	EAWAG isolate GM17	Salinity vs pH & Alkalinity	0.1391	-0.2643	0.5424	0.454	ns
Pediastrum boryanum	EAWAG isolate GM20	pH & Alkalinity vs Control	-2.2767	-3.2491	-1.3042	0.002	**
Pediastrum boryanum	EAWAG isolate GM20	Salinity vs Control	-1.2439	-1.7234	-0.7645	5.00E-04	***

Pediastrum boryanum	EAWAG isolate GM20	Salinity vs pH & Alkalinity	1.0328	0.0646	2.0009	0.041	*
Pediastrum boryanum	SAG 87.81	pH & Alkalinity vs Control	-1.8061	-2.2107	-1.4015	4.00E-04	***
Pediastrum boryanum	SAG 87.81	Salinity vs Control	-0.4287	-0.5532	-0.3043	1.00E-04	***
Pediastrum boryanum	SAG 87.81	Salinity vs pH & Alkalinity	1.3773	0.9682	1.7865	0.001	***
Pediastrum duplex	EAWAG isolate GM22	pH & Alkalinity vs Control	-4.3651	-4.8209	-3.9093	0	****
Pediastrum duplex	EAWAG isolate GM22	Salinity vs Control	-2.943	-3.3728	-2.5132	0	****
Pediastrum duplex	EAWAG isolate GM22	Salinity vs pH & Alkalinity	1.4221	0.9526	1.8916	2.00E-04	***
Pediastrum duplex	SAG 261-2	pH & Alkalinity vs Control	-1.3662	-2.2735	-0.4588	0.016	*
Pediastrum duplex	SAG 261-2	Salinity vs Control	-0.2262	-0.3261	-0.1264	0.003	**
Pediastrum duplex	SAG 261-2	Salinity vs pH & Alkalinity	1.1399	0.2406	2.0393	0.026	*
Planktothrix rubescens	NIVA-CYA 619	pH & Alkalinity vs Control	-1.3883	-1.6417	-1.1349	0	****
Planktothrix rubescens	NIVA-CYA 619	Salinity vs Control	-0.4429	-0.6871	-0.1988	0.004	**
Planktothrix rubescens	NIVA-CYA 619	Salinity vs pH & Alkalinity	0.9454	0.6655	1.2252	1.00E-04	***
Poterioochromonas malhamensis	CCAP 933/1C	pH & Alkalinity vs Control	-2.4024	-2.9561	-1.8487	2.00E-04	***
Poterioochromonas malhamensis	CCAP 933/1C	Salinity vs Control	0.6003	0.039	1.1616	0.041	*
Poterioochromonas malhamensis	CCAP 933/1C	Salinity vs pH & Alkalinity	3.0027	2.7196	3.2858	0	****
Scenedesmus acuminatus	SAG 38.81	pH & Alkalinity vs Control	-3.8373	-6.0662	-1.6085	0.011	*
Scenedesmus acuminatus	SAG 38.81	Salinity vs Control	-0.2998	-0.3676	-0.2319	2.00E-04	***
Scenedesmus acuminatus	SAG 38.81	Salinity vs pH & Alkalinity	3.5375	1.3071	5.768	0.014	*
Scenedesmus armatus	EAWAG isolate GM28	pH & Alkalinity vs Control	-0.366	-0.4498	-0.2822	3.00E-04	***
Scenedesmus armatus	EAWAG isolate GM28	Salinity vs Control	-0.045	-0.0786	-0.0114	0.015	*
Scenedesmus armatus	EAWAG isolate GM28	Salinity vs pH & Alkalinity	0.3211	0.2367	0.4054	5.00E-04	***
Scenedesmus ellipticus	EAWAG isolate GM25	pH & Alkalinity vs Control	-0.6637	-0.8198	-0.5076	1.00E-04	****
Scenedesmus ellipticus	EAWAG isolate GM25	Salinity vs Control	-0.4089	-0.5892	-0.2285	0.001	***
Scenedesmus ellipticus	EAWAG isolate GM25	Salinity vs pH & Alkalinity	0.2548	0.0908	0.4188	0.009	**
Staurastrum punctulatum	SAG 679-1	pH & Alkalinity vs Control	-3.5085	-3.8099	-3.2071	0	****
Staurastrum punctulatum	SAG 679-1	Salinity vs Control	-3.542	-3.9212	-3.1627	0	****
Staurastrum punctulatum	SAG 679-1	Salinity vs pH & Alkalinity	-0.0335	-0.4151	0.3481	0.96	ns

Synechococcus elongatus	EAWAG isolate GM48	pH & Alkalinity vs Control	-0.9615	-1.0781	-0.845	0	****
Synechococcus elongatus	EAWAG isolate GM48	Salinity vs Control	0.4462	0.3708	0.5216	2.00E-04	***
Synechococcus elongatus	EAWAG isolate GM48	Salinity vs pH & Alkalinity	1.4077	1.2976	1.5179	0	****
Synechocystis sp.	PCC 6803	pH & Alkalinity vs Control	0.1364	0.0261	0.2467	0.026	*
Synechocystis sp.	PCC 6803	Salinity vs Control	0.2517	0.1914	0.312	1.00E-04	****
Synechocystis sp.	PCC 6803	Salinity vs pH & Alkalinity	0.1153	0.0081	0.2225	0.039	*
Tetraedron minimum	SAG 44.81	pH & Alkalinity vs Control	-1.6503	-1.7548	-1.5458	0	****
Tetraedron minimum	SAG 44.81	Salinity vs Control	-0.6059	-0.692	-0.5198	0	****
Tetraedron minimum	SAG 44.81	Salinity vs pH & Alkalinity	1.0444	0.9344	1.1543	0	****

B. Supplementary Figures

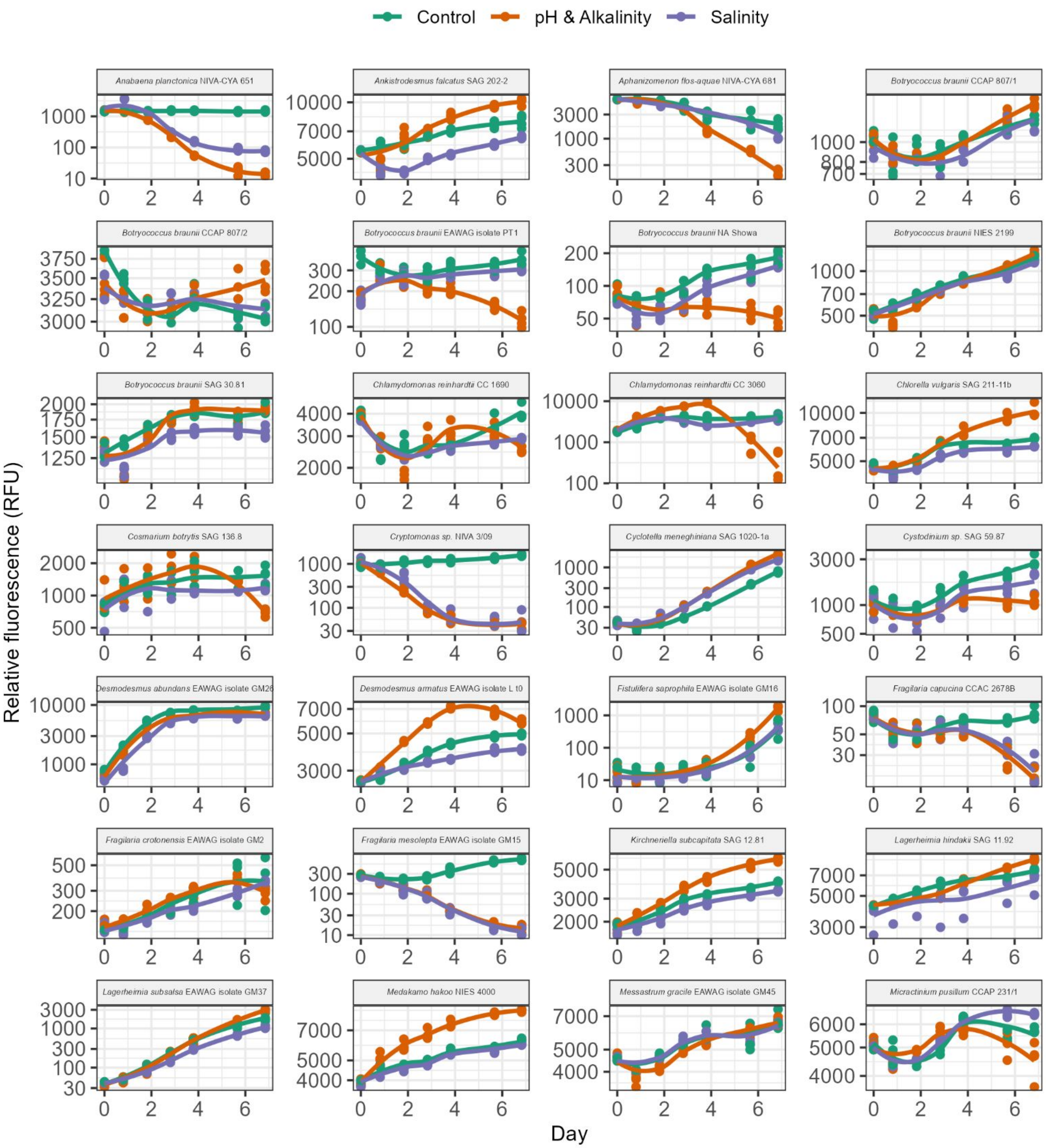

Figure S1. Growth curves of all microalgae strains in Phase 1 (i.e., the acclimation phase to a mild increase in pH and alkalinity – pH 8.5 and 25 mM of alkalinity added as carbonates). Points show the relative fluorescence values of each replicate and lines show loess fits. Chl-a fluorescence (ex/em at 445/685 nm) is shown for all strains except the cyanobacteria, for which phycocyanin fluorescence (ex/em at 586/647 nm) is shown. Continued on next page.

Figure S1 continued:

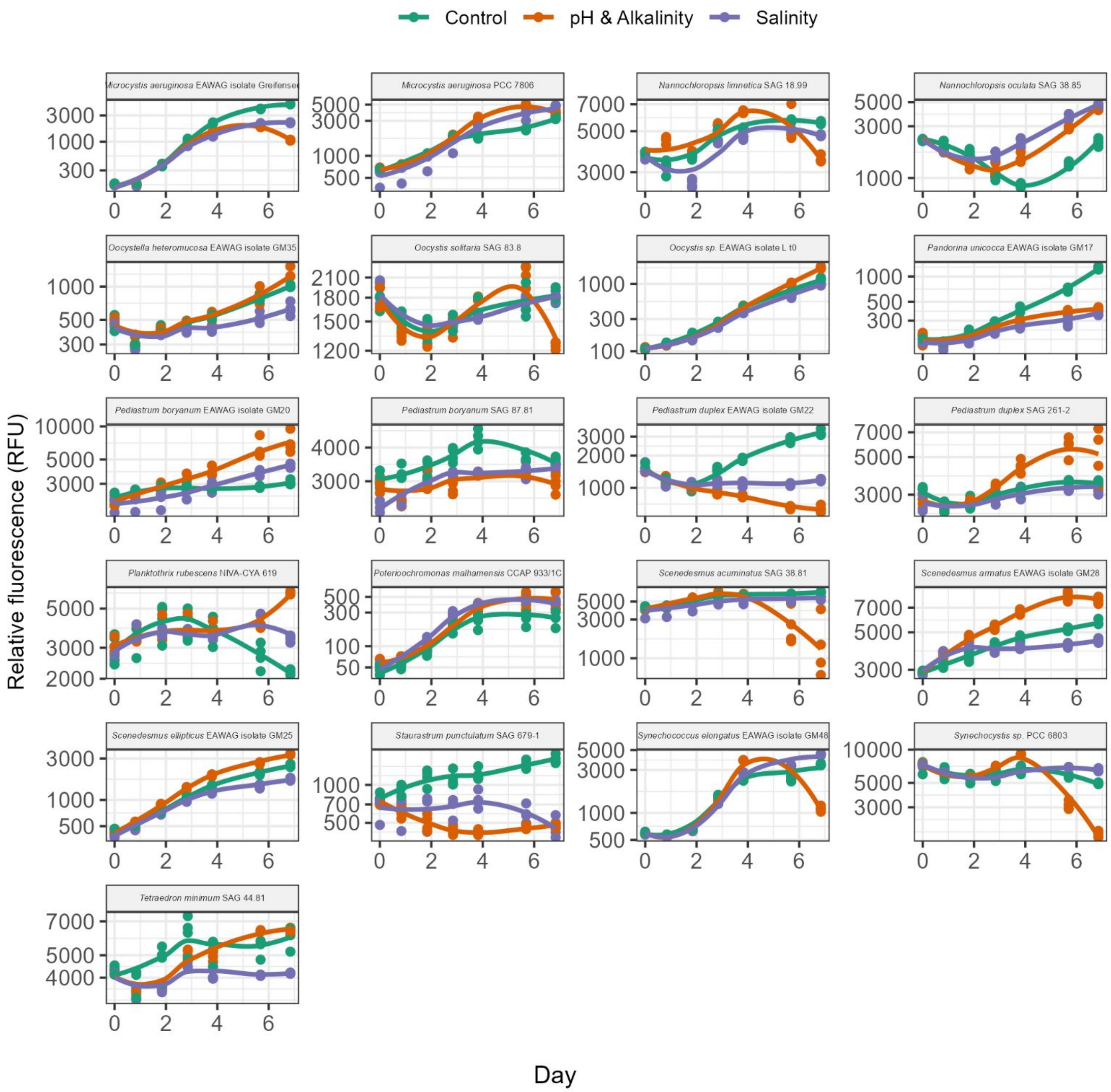

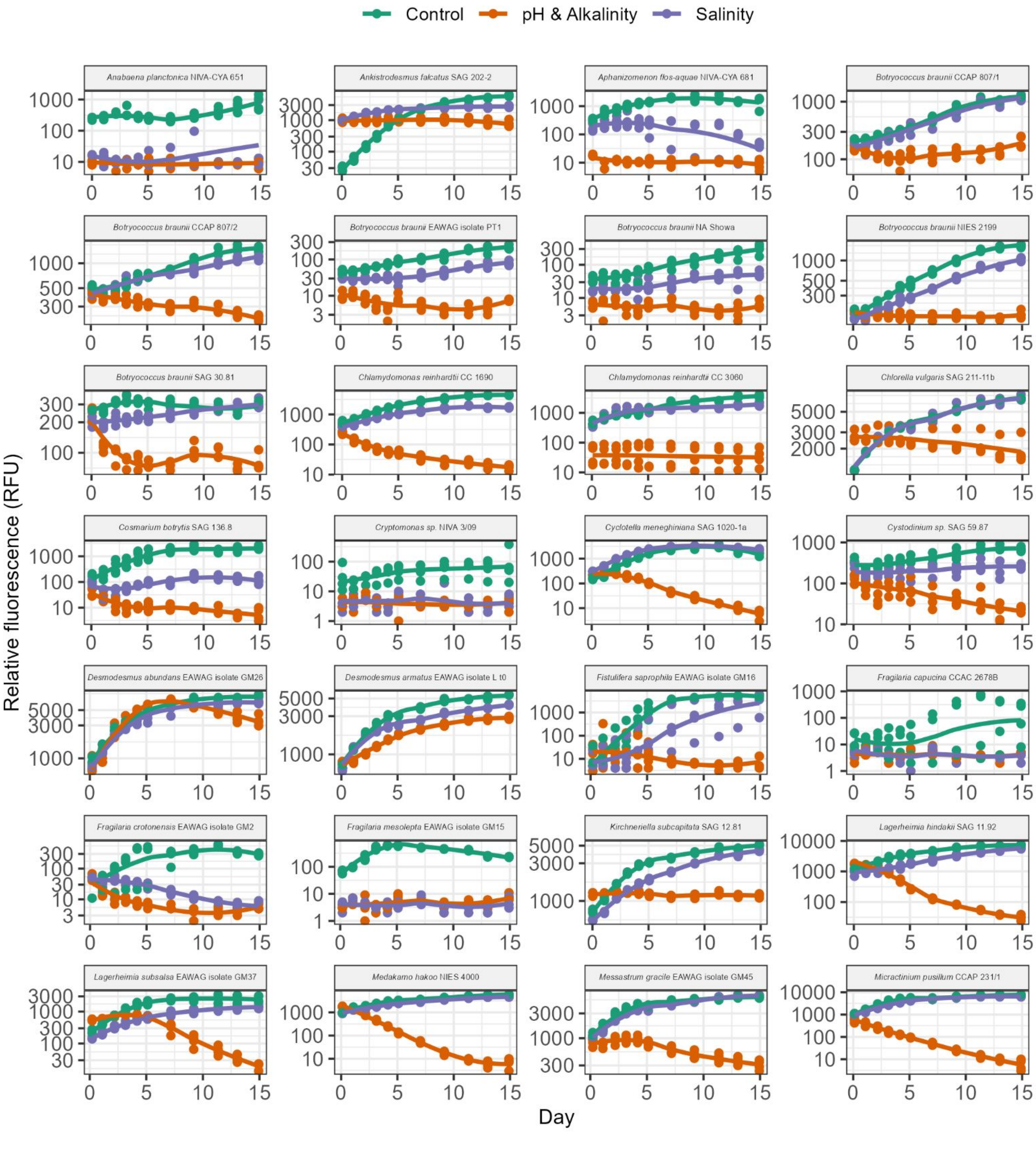

Figure S2. Growth curves of all microalgae strains in Phase 2 (i.e., the screening in high pH and alkalinity – pH 10 and 75 mM of alkalinity added as carbonates). Points show the relative fluorescence values of each replicate and lines show loess fits. Chl-a fluorescence (ex/em at 445/685 nm) is shown for all strains except the cyanobacteria, for which phycocyanin fluorescence (ex/em at 586/647 nm) is shown. Continued on next page.

Figure S2 continued:

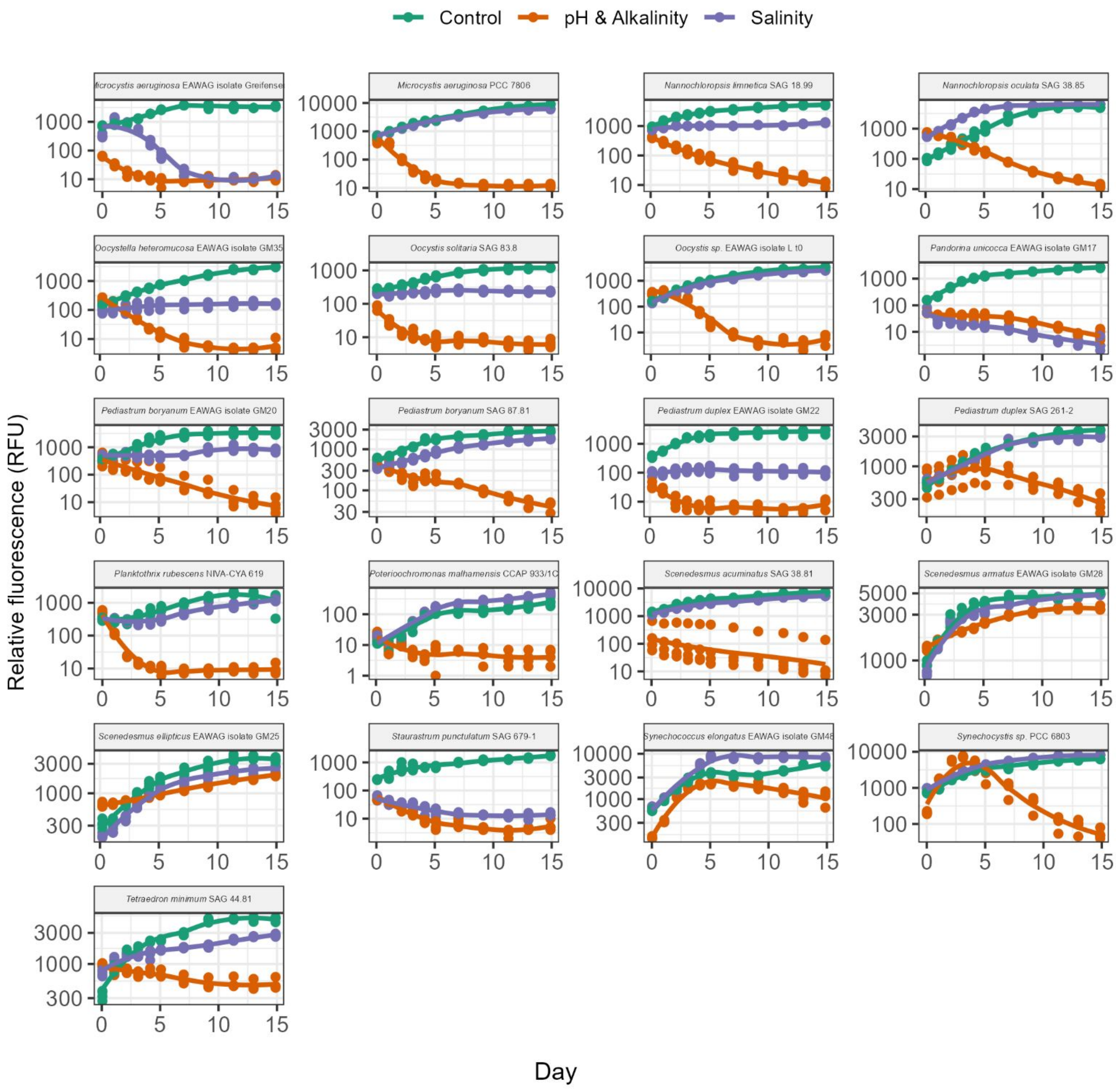

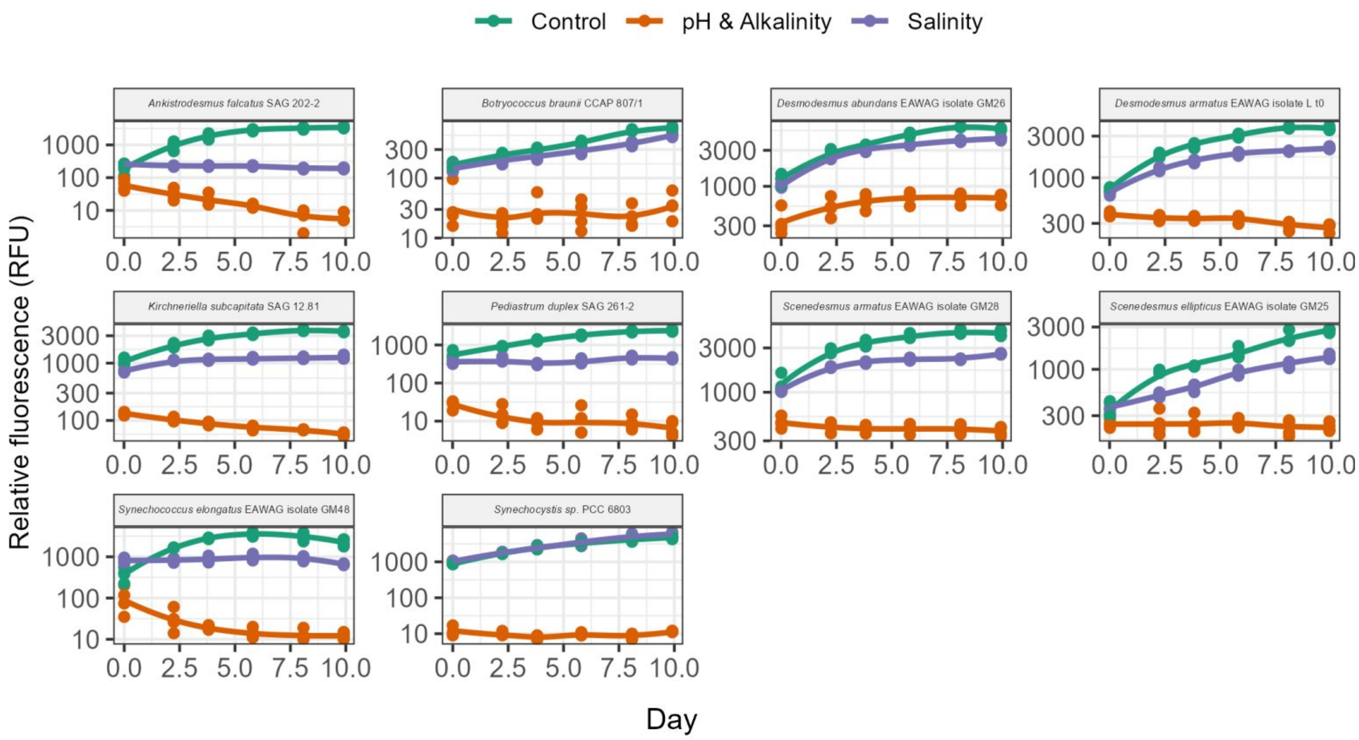

Figure S3. Growth curves of all microalgae strains in Phase 3 (i.e., the screening in extreme pH and alkalinity – pH 10 and 150 mM of alkalinity added as carbonates). Points show the relative fluorescence values of each replicate and lines show loess fits. Chl-a fluorescence (ex/em at 445/685 nm) is shown for all strains except the cyanobacteria, for which phycocyanin fluorescence (ex/em at 586/647 nm) is shown.

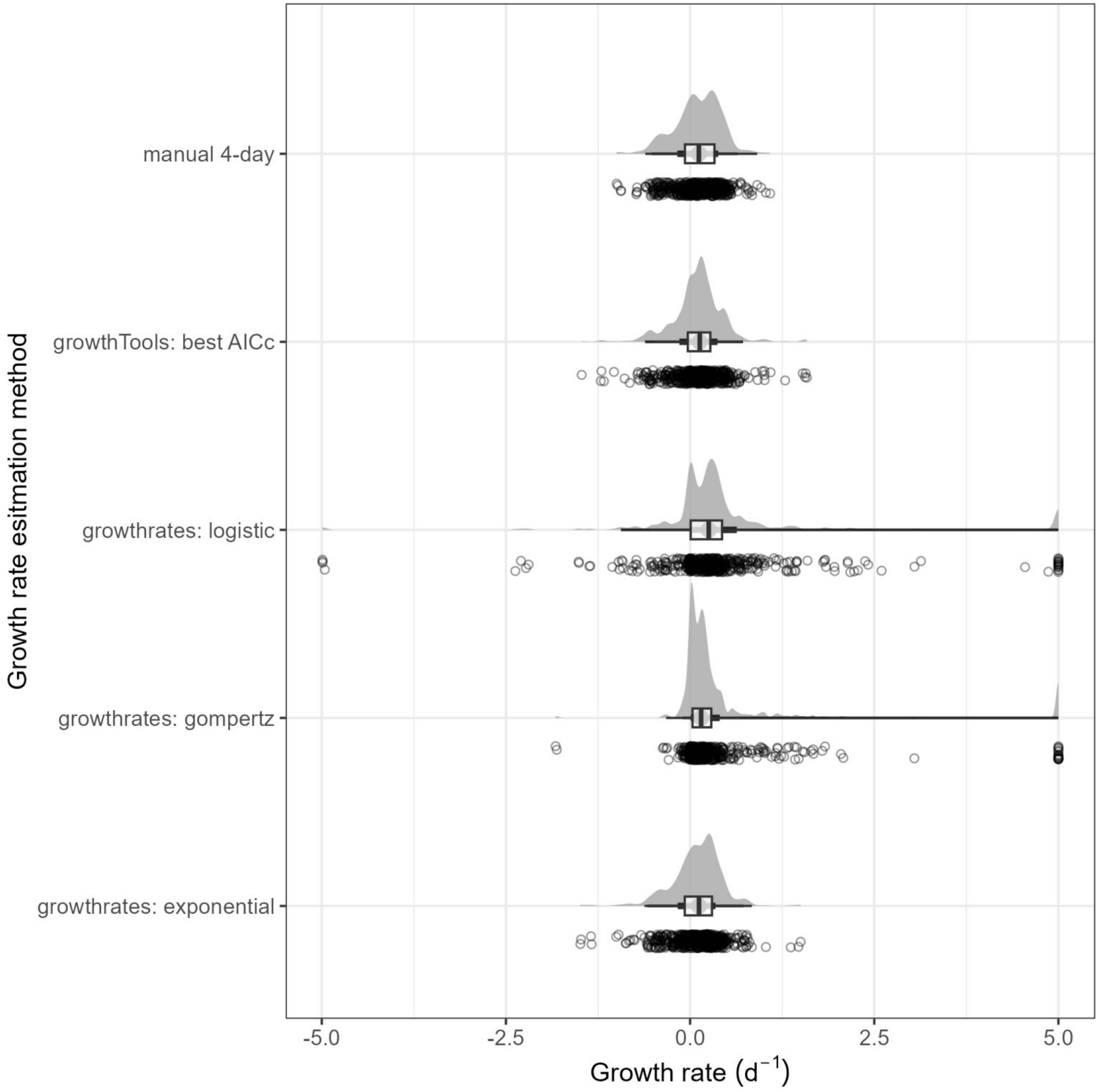

Figure S4. Distribution of growth rate estimates in Phase 2 using five different methods. Points show estimates for each experimental unit, density plots show smoothed distributions, and boxplots show default statistics (median, 25th to 75th percentile, and extreme data points within 1.5x the interquartile range). Logistic and Gompertz models often provided unrealistically high estimates which hit the maximum value allowed (µmax of 5), whereas the manual approach, growthTools, and growthrates (exponential) models all provided similar results.

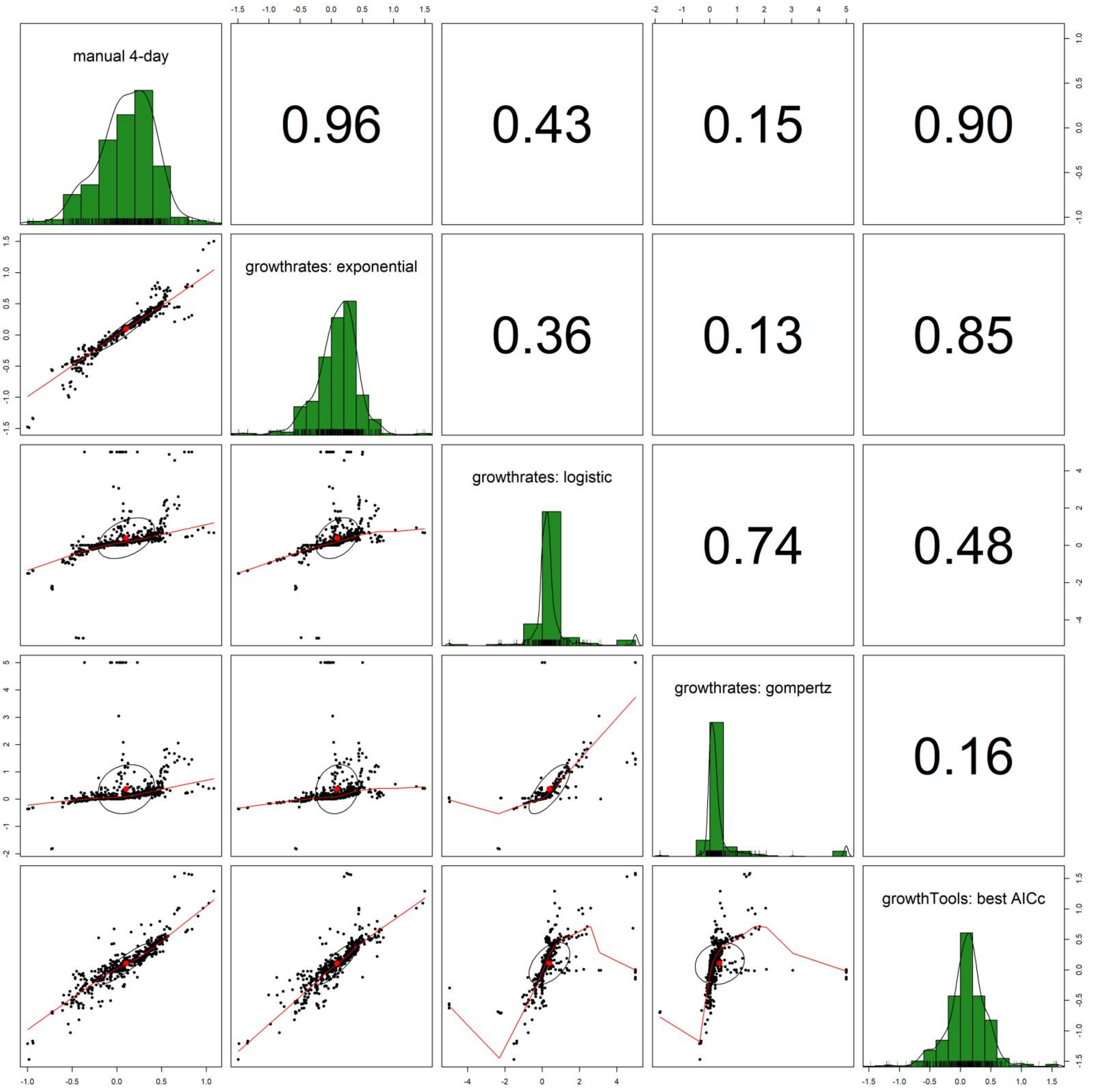

Figure S5. Correlation plots showing similarities among growth rate estimation methods applied for Phase 2. Diagonals show histograms of growth rates, numbers in upper right indicate Pearson correlation coefficients, and lower left figures plot growth rate estimate methods against each other for all 588 experimental units, with red lines showing loess fits to the data.

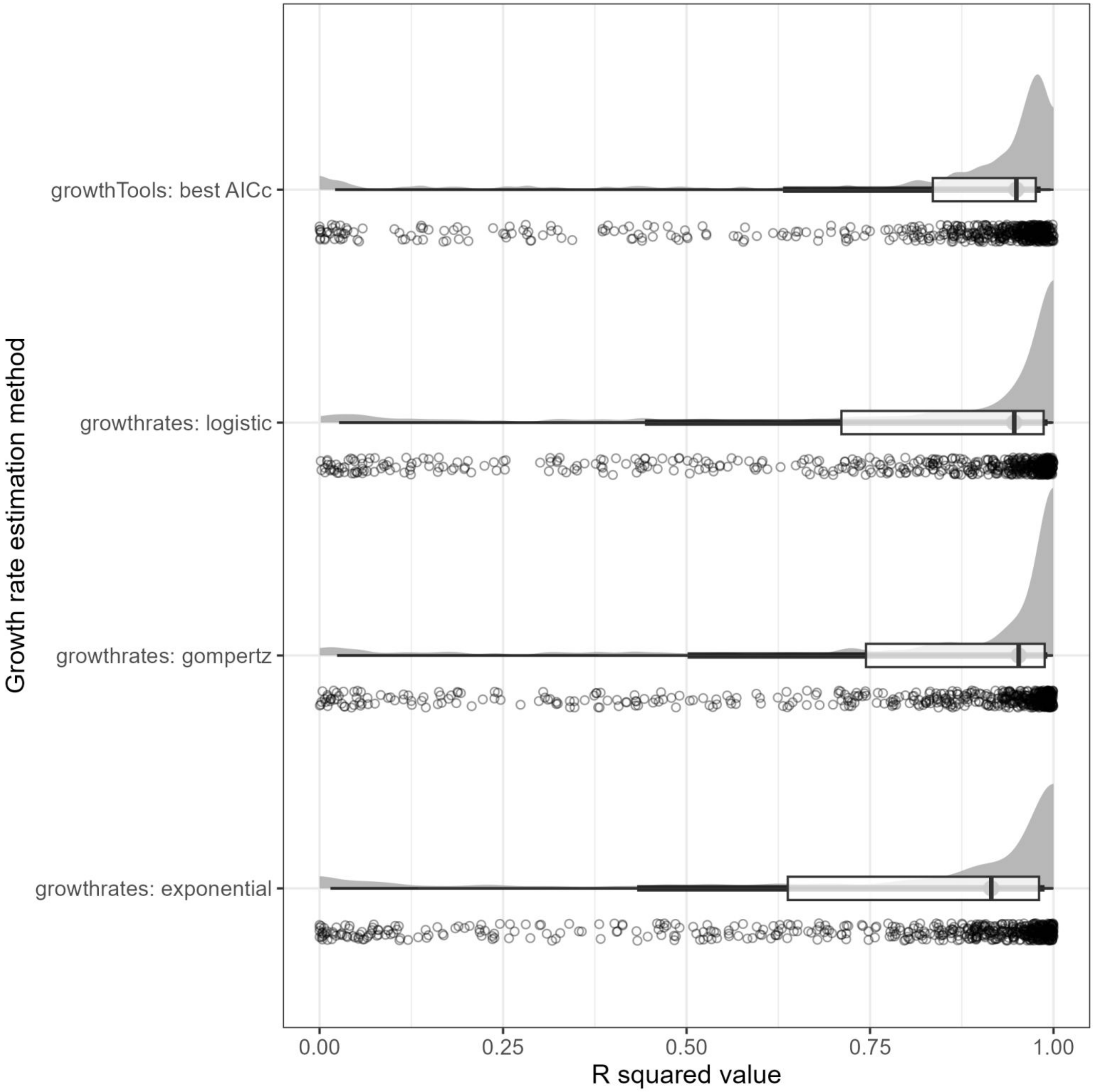

Figure S6. Distribution of R^2 values for the four different growth models assessed in Phase 2. Points show estimates for each experimental unit, density plots show smoothed distributions, and boxplots show default statistics (median, 25th to 75th percentile, and extreme data points within 1.5x the interquartile range).

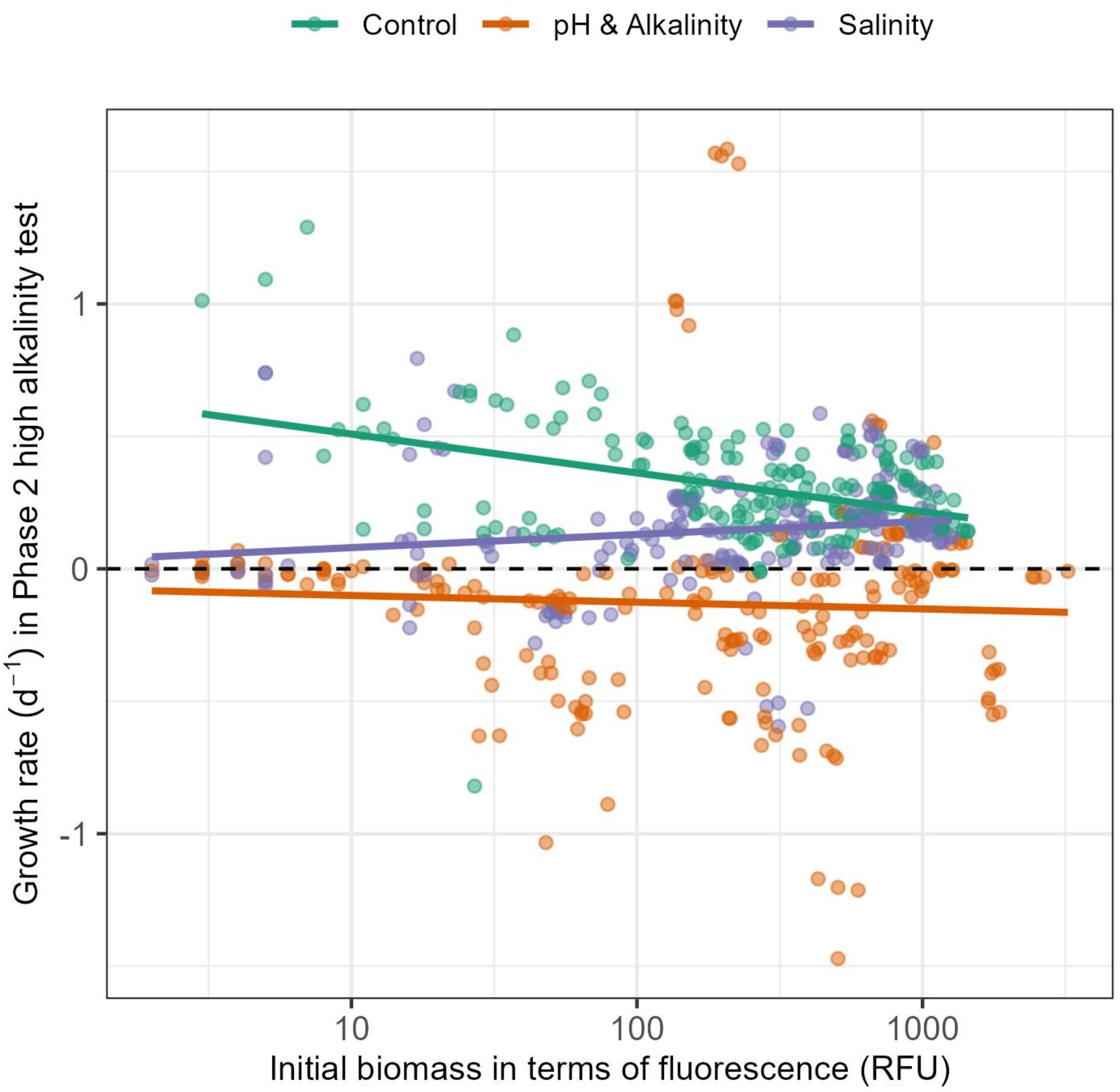

Figure S7. Effects of the initial inoculation density (in terms of chlorophyll fluorescence) on growth rates in Phase 2. An additive linear model shows no overall effect of initial density on growth rate ($p = 0.12$); however, a model including an interaction term between the media treatment and initial density shows that there is a significant interactive effect; i.e., initial density effects depend on the medium type ($F_{5,582} = 48.7$, p-value for interaction terms < 0.007). When performing linear regressions independently for each medium type, regression parameters are as follows: Control: $\mu max = -0.064 \times$ log_initial_density + 0.66 ($R^2 = 0.16$); pH & alkalinity: $\mu max = -0.011 \times$ log_initial_density – 0.075 ($R^2 = 0.002$); Salinity: $\mu max = 0.022 \times$ log_initial_density + 0.030 ($R^2 = 0.026$). In summary, this analysis of the effect of initial densities shows no effect for the pH & alkalinity treatment, a small positive effect for the salinity treatment, and a negative effect for the control treatment. Therefore, growth rates may be underestimated for controls when transfers resulted in relatively initial high densities, but there is no evidence to suggest that growth in either the pH & alkalinity or salinity treatments was inhibited due to relatively high initial densities.

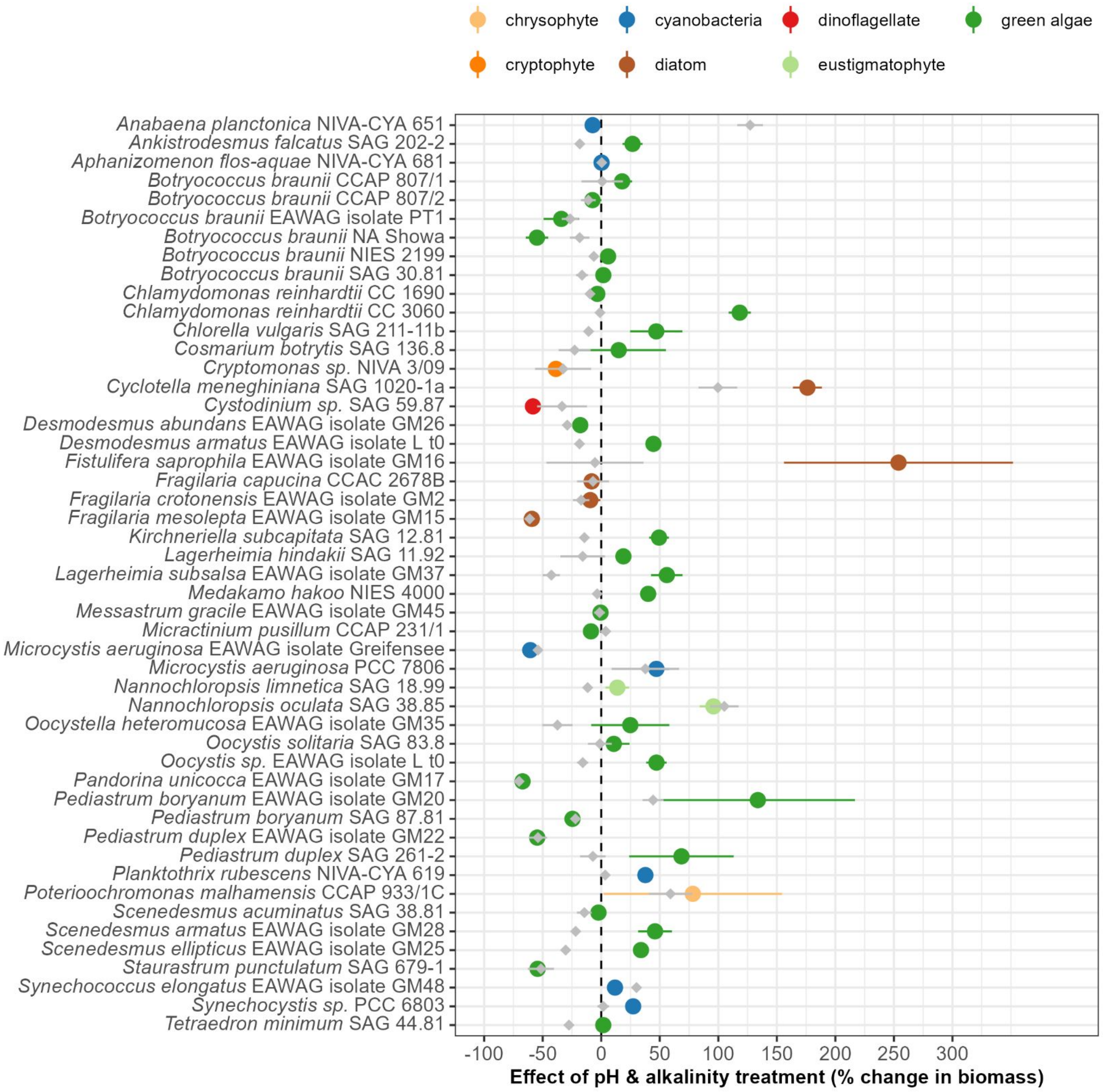

Figure S8. Same data as Figure 1 but ordered alphabetically by strain. Effects of the pH and alkalinity treatment on algal biomass concentration during Phase 1 (i.e., during the 7-day acclimation phase to a mild increase in initial pH and alkalinity: pH 8.5 and 25 mM of alkalinity added as carbonates). Colored circles and corresponding lines represent the mean and 95% confidence interval of the pH/alkalinity treatment effect in the form of maximum biomass production estimated as *Effect size = (treatment max. fluorescence - mean control max. fluorescence)/(mean control max. fluorescence)*. Gray diamonds and lines show the mean and 95% CI effect of salinity (2.1 g/L NaCl during Phase 1) for each strain. Values above zero indicate a positive effect of the treatment relative to control, zero indicates no effect, and negative values indicate a detrimental effect of the pH/alkalinity (or salinity) treatment.

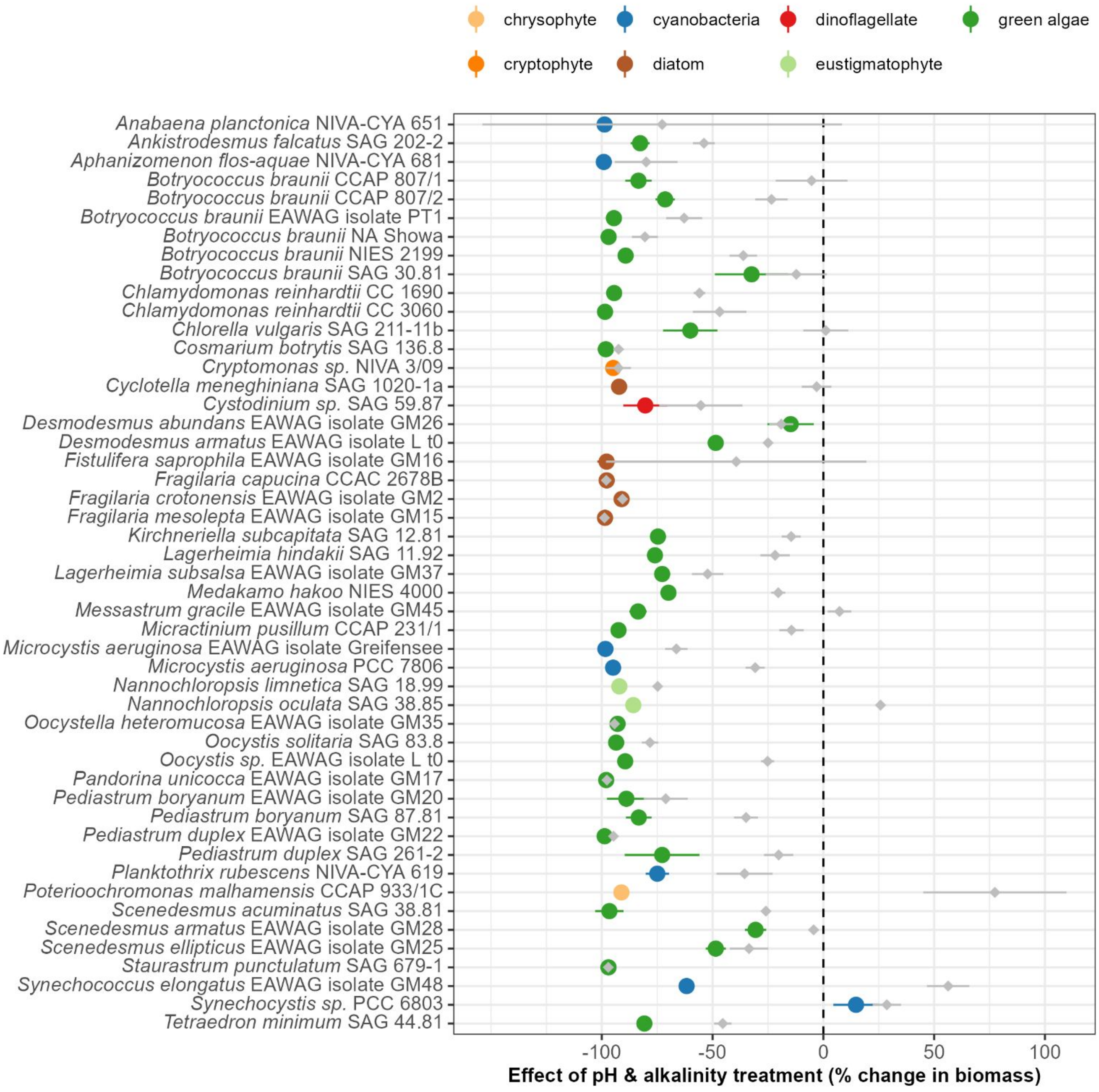

Figure S9. Same data as Figure 3 but ordered alphabetically by strain. Effects of the pH and alkalinity treatment on algal biomass concentration during Phase 2 (i.e., the 15-day screening in high pH and alkalinity – pH 10 and 75 mM of alkalinity added as carbonates). Colored circles and corresponding lines represent the mean and 95% confidence interval of the pH/alkalinity treatment effect in the form of maximum biomass production estimated as *Effect size = (treatment max. fluorescence - mean control max. fluorescence)/(mean control max. fluorescence)*. Gray diamonds and lines show the mean and 95% CI effect of salinity (4.75 g/L NaCl during Phase 2) for each strain. Values above zero indicate a positive effect of the treatment relative to control, zero indicates no effect, and negative values indicate a detrimental effect of the pH/alkalinity (or salinity) treatment.

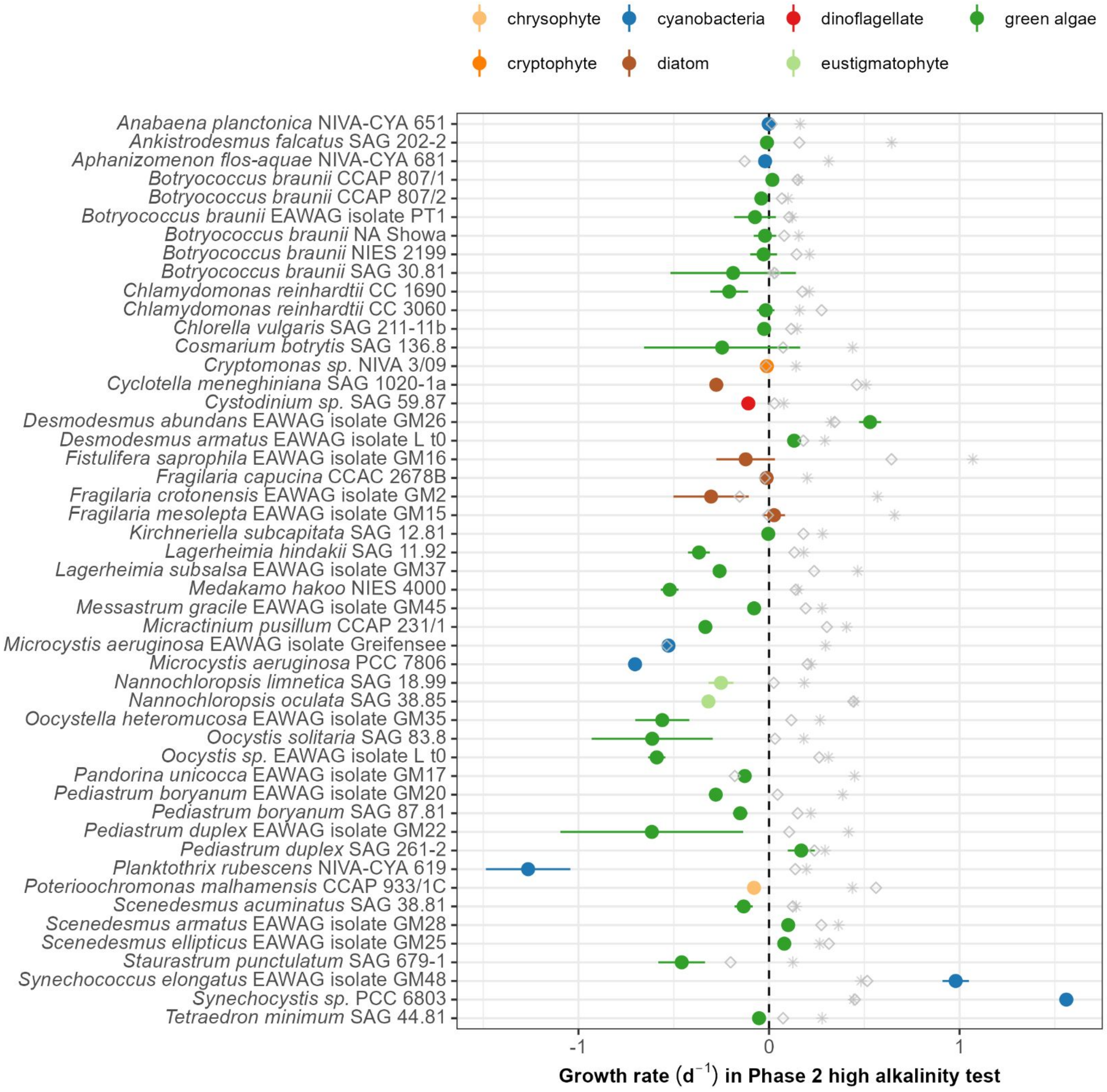

Figure S10. Same data as Figure 4 but ordered alphabetically by strain. Growth rates of each algal strain in the high pH and alkalinity screening (Phase 2, 15-day screening at pH 10 and 75 mM of alkalinity added as carbonates) estimated using the R package `growthTools`. Colored circles and corresponding lines represent the mean and 95% confidence interval of growth rate in high pH and alkalinity; gray diamonds show the average growth rates of the salinity treatments (4.75 g/L NaCl), and asterisks show the average growth rates in the control treatment.
